# Engineering *Yarrowia lipolytica* to Enhance the Production of Malonic Acid via Malonyl‐CoA Pathway at High Titer

**DOI:** 10.1002/advs.202411665

**Published:** 2025-02-07

**Authors:** Qun Yang, Mengzhen Tian, Ping Dong, Yunying Zhao, Yu Deng

**Affiliations:** ^1^ School of Biotechnology and Key Laboratory of Industrial Biotechnology of Ministry of Education Jiangnan University 1800 Lihu Road Wuxi Jiangsu 214122 China; ^2^ National Engineering Research Center of Cereal Fermentation and Food Biomanufacturing Jiangnan University 1800 Lihu Road Wuxi Jiangsu 214122 China; ^3^ Jiangsu Provincial Research Center for Bioactive Product Processing Technology Jiangnan University 1800 Lihu Road Wuxi Jiangsu 214122 China

**Keywords:** acetyl‐CoA, Malonic acid (MA), malonyl‐CoA hydrolase, malonyl‐CoA, *Yarrowia lipolytica*

## Abstract

Malonic acid (MA) is a high‐value‐added chemical with significant applications in the polymers, pharmaceutical, and food industries. Microbial production of MA presents enzyme inefficiencies, competitive metabolic pathways, and dispersive carbon flux, which collectively limit its biosynthesis. Here, the non‐conventional oleaginous yeast *Yarrowia lipolytica* is genetically engineered to enhance MA production. Initially, the malonyl‐CoA pathway, comprising a malonyl‐CoA hydrolase from *Saccharomyces cerevisiae*, is confirmed as the most efficient for MA production in *Y. lipolytica*. To further enhance MA production, two novel malonyl‐CoA hydrolases exhibiting higher activity than the hydrolase from *S. cerevisiae*, are identified from *Y. lipolytica* and *Fusarium oxysporum*, respectively. The introduction of the malonyl‐CoA hydrolase from *F. oxysporum* increases the MA titer to 6.3 g L^−1^. Subsequently, advanced metabolic engineering strategies are performed to ensure a sufficient flux of the precursors acetyl‐CoA and malonyl‐CoA for MA production, resulting in a production of 13.8 g L^−1^ MA in shaking‐flasks. Finally, by employing the fermentation conditions and feeding strategies, a maximum concentration of 63.6 g L^−1^ of MA is achieved at 156 h with a productivity of 0.41 g L^−1^ h^−1^ in fed‐batch fermentation. This study provides a new way for engineering *Y. lipolytica* to enhance MA production at high titer.

## Graphical abstract

The non‐conventional oleaginous yeast *Y. lipolytica* has been genetically engineered to produce malonic acid via malonyl‐CoA pathway through introducing an efficient malonyl‐CoA hydrolase. Advanced metabolic engineering and optimization of fermentation conditions resulted in a malonic acid production yield of 19.1 g L^−1^ in shaking‐flasks. Further optimization through fed‐batch fermentation techniques led to a maximum malonic acid concentration of 63.6 g L^−1^.

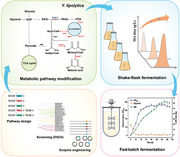

## 1 Introduction

Malonic acid (MA) is an organic dicarboxylic acid with a wide range of applications in food, pharmaceuticals, manufacturing, and chemical industries. As a platform chemical, MA serves as a precursor for numerous flavors, fragrances, and pharmaceuticals, including cinnamic acid, 3,4,5‐trimethoxycinnamic acid, and γ‐nonanolactone.^[^ ^1^ ^]^ Additionally, MA can also be used in manufacturing industries, particularly in electronics,^[^ ^2^ ^]^ specialty solvents, and polymer cross‐linking.^[^ ^3^ ^]^ For these applications, MA has been listed as one of the top 30 chemicals that can be produced from biomass by the United States Department of Energy.^[^ ^4^ ^]^ Currently, MA is predominantly produced industrially by chemical synthesis, specifically through the hydrolysis of diethyl malonate and cyanoacetic acid.^[^ ^5^ ^]^ However, the hydrolysis of diethyl malonate is prone to reversible reactions, MA undergoes decarboxylation, decomposing into acetic acid, water and carbon dioxide by heat under high temperature conditions, which results in low product yield. Conversely, the hydrolysis of cyanoacetic acid is a complex process that generates impurities, thereby reducing the purity of MA. Therefore, there is an urgent need for the development of a sustainable, efficient and environmentally friendly method for the production of MA.

Biological production of MA has been demonstrated in the microorganisms of *Escherichia Coli*, *Myceliophthora thermophila*, and *Saccharomyces cerevisiae*.^[^ ^6^ ^]^ At present, MA can be synthesized via three intermediates: β‐alanine,^[^ ^6a^ ^]^ oxaloacetate,^[^ ^6c^ ^]^ and malonyl‐CoA.^[^ ^6b^ ^]^ The β‐alanine pathway was constructed to produce MA from β‐alanine in *E. coli* by introducing the β‐alanine pyruvate transaminase encoded by *pa0132* gene from *Pseudomonas aeruginosa* and overexpressing the *E. coli* succinate semialdehyde dehydrogenase encoded by *yneI* gene, resulting in 3.60 g L^−1^ by fed‐batch fermentation.^[^ ^6a^ ^]^ Then, a novel MA synthetic pathway was designed and constructed in *M. thermophila* using oxaloacetate as a precursor. This pathway involved the conversion of oxaloacetate to malonate‐semialdehyde via oxaloacetate dehydrogenase (Mdc), followed by the reduction of malonate‐semialdehyde to MA by the dehydrogenase YneI, which produced only 42.5 mg L^−1^ MA.^[^ ^6c^ ^]^ Given the inherent robustness of the budding yeast *S. cerevisiae*, such as its resistance to acidic conditions and lake of phage contamination, the β‐alanine pathway was first ported from *E. coli* to *S. cerevisiae* to facilitate the production of MA in our previous study.^[^ ^7^ ^]^ However, the maximum titer of MA achieved was only 91.5 mg L^−1^, which was significantly lower than that observed in *E. coli*. It has been reported that the native gene encoding 3‐hydroxyisobutyryl‐CoA hydrolase in *S. cerevisiae* could be mutated to exhibit malonyl‐CoA hydrolase activity and catalyze the conversion of malonyl‐CoA to MA.^[^ ^6b^ ^]^ To enhance the production of MA, we next constructed the malonyl‐CoA pathway by targeting the mitochondrial 3‐hydroxyisobutyryl‐CoA hydrolase gene *EHD3* from *S. cerevisiae* to the cytoplasm and mutating its active sites to obtain malonyl‐CoA hydrolase activity.^[^ ^8^ ^]^ This genetic modification, combined with the efforts of improving the precursor supply of malonyl‐CoA and optimizing the fermentation conditions, the MA titer was increased to 1.6 g L^−1^ after the fed‐batch fermentation.^[^ ^8^ ^]^ However, this MA titer remains significantly lower than that achieved via the β‐alanine pathway *E. coli* in the previous study,^[^ ^6a^ ^]^ indicating that the activity of malonyl‐CoA hydrolase might be not high enough for MA production. In addition, using *S. cerevisiae* as a production host usually challenged with ethanol accumulation,^[^ ^9^ ^]^ leading to the scattered carbon flux and limiting MA production. Therefore, employing a more efficient malonyl‐CoA hydrolase and an attractive host with abundant malonyl‐CoA flux could potentially enhance MA production to a higher level.

*Yarrowia lipolytica* is a “generally regarded as safe” (GRAS) yeast,^[^ ^10^ ^]^ which has several advantages, including the ability to metabolize various carbon sources, excellent acid tolerance, elevated levels of acetyl‐CoA and malonyl‐CoA, a broad pH tolerance range, the capacity to achieve high cell densities and being unaffected by glucose repression.^[^ ^11^ ^]^ Recently, the advancements in genetic tools have highlighted the significant potential of *Y. lipolytica* to produce various malonyl‐CoA‐derived products.^[^ ^12^ ^]^ *Y. lipolytica* has been effectively engineered and optimized as a microbial cell factory for the production of lipids,^[^ ^13^ ^]^ natural product^[^ ^14^ ^]^ and terpenoids,^[^ ^15^ ^]^ such as squalene,^[^ ^16^ ^]^ farnesene,^[^ ^17^ ^]^ germacrene A,^[^ ^18^ ^]^ and carotenoids.^[^ ^19^ ^]^ This is largely attributed to the robust activity of its tricarboxylic acid (TCA) cycle and acetyl‐CoA metabolism. As a prototypical oleaginous yeast, *Y. lipolytica* exhibits exceptional lipid accumulation capabilities, achieving a lipid titer of 72.7 g L^−1^ and an oil content of 81.4% in bioreactor settings at an industrial scale.^[^ ^20^ ^]^ Furthermore, the biosynthetic pathway for polydatin has been successfully established in *Y. lipolytica*, yielding 6.88 g L^−1^, which represents the highest reported level of polydatin production.^[^ ^21^ ^]^ Additionally, the synthesis of another malonyl‐CoA derivative, 3‐hydroxypropionic acid (3‐HP), has been efficiently accomplished, with yields reaching 1.128 g L^−1^ in shake flask fermentation and 16.23 g L^−1^ in fed‐batch fermentation using the recombinant strain Po1f‐NC‐14.^[^ ^22^ ^]^ These achievements are closely linked to the high supply of acetyl‐CoA and malonyl‐CoA precursors of *Y. lipolytica*.^[^ ^23^ ^]^

In this study, we aim to develop a more efficient *Y. lipolytica* cell factory for MA production. The efficiencies of three MA synthesis pathways of malonyl‐CoA pathway, malonyl‐CoA and malonate‐semialdehyde pathway, oxaloacetate and malonate‐semialdehyde pathway are evaluated. The malonyl‐CoA pathway is identified as the most effective pathway for MA production. Subsequently, two novel malonyl‐CoA hydrolases exhibiting higher activities are identified from *Y. lipolytica* and *Fusarium oxysporum*, respectively, and utilized for MA production. To enhance the precursor supply of acetyl‐CoA and malonyl‐CoA, their competing pathways are further inhibited and the key enzymes are overexpressed. Ultimately, through the combined efforts of fermentation optimization and fed‐batch fermentation, an MA titer of 63.6 g L^−1^ is achieved. This represents the highest titer reported to date and constitutes a significant breakthrough in the biosynthesis of MA.

## 2 Results

### 2.1 Investigating Optimal Biosynthetic Pathway for MA Production in *Y. lipolytica*

**Figure 1:**
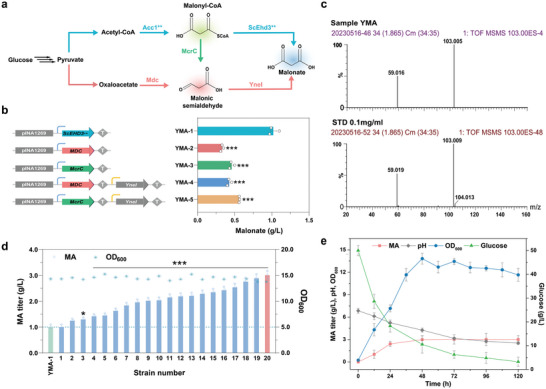
Biosynthesis of MA by the yeast *Y. lipolytica*. a) The design of three distinct biosynthetic pathways for MA production. b) Comparison of the MA production by three pathways. The asterisks show statistically significant differences relative to the YMA‐1 strain. c) Identification of MA production by liquid chromatography‐mass spectrometry (LC‐MS). d) Evaluation of the MA production levels of the 20 screened colonies. The asterisks show statistically significant differences from ScEhd3** single‐copy number integrated strain. e) Analysis of the growth, MA production, pH value, and glucose consumption of the strain YMA‐6 in shake‐flask fermentation. The data were presented as mean values ± SD from three independent biological replicates (n = 3). The error bars represent the standard deviation (s.d.). Statistical significance was evaluated using one‐way analysis of variance (ANOVA). The asterisks of *, ** and *** denote *p* < 0.05, *p* < 0.01 and *p* < 0.001, respectively.

To produce MA in *Y. lipolytica*, a malonyl‐CoA pathway and two malonate‐semialdehyde pathways were constructed (**Figure** **1**a). First, the malonyl‐CoA pathway was established by overexpressing the malonyl‐CoA hydrolase derived from the 3‐hydroxyisobutyryl‐CoA hydrolase Ehd3** of *S. cerevisiae*, incorporating two specific mutations, F121I and E124S.^[^ ^8^ ^]^ It has been reported that 3‐hydroxypropionic acid (3‐HP) can be effectively synthesized from malonate‐semialdehyde.^[^ ^22^ ^]^ Given the synthesis of MA via the malonate‐semialdehyde pathway has not been investigated in yeast, we focus on two malonate‐semialdehyde pathways by using oxaloacetate or malonyl‐CoA as a precursor, respectively. Then, the oxaloacetate and malonate‐semialdehyde pathway (Mdc pathway) was constructed in *Y. lipolytica* by introducing the oxaloacetate decarboxylase (Mdc) from *O. parapolymorpha* and the malonate‐semialdehyde dehydrogenase Yne1 from *E. coli*. To construct the malonyl‐CoA and malonate‐semialdehyde pathway (McrC pathway), the C‐terminal fragment (McrC, amino acids 550–1219) of malonyl‐CoA reductase (Mcr) from *C. aurantiacus* which can reduce malonyl‐CoA to malonate‐semialdehyde,^[^ ^24^ ^]^ and the malonate‐semialdehyde dehydrogenase Yne1 from *E. coli*, were both overexpressed.

The malonyl‐CoA hydrolase of Ehd3** from *S. cerevisiae*, the oxaloacetate decarboxylase Mdc from *O. parapolymorpha*, the malonyl‐CoA reductase McrC from *C. aurantiacus* as well as the malonate‐semialdehyde dehydrogenase Yne1 from *E. coli* was cloned into the single‐copy number integrated plasmid of pINA1269 and expressed using the hp4d promoter and *XPR2* terminator, respectively. These plasmids were subsequently linearized and integrated to the genome of Po1f strain. Following shake flask fermentation, the YMA‐1 strain harboring the malonyl‐CoA pathway, achieved a maximum MA titer of 1.0 g L^−1^ (Figure 1b,c). Interestingly, the YMA‐2 strain expressing only the oxaloacetate decarboxylase Mdc and the YMA‐3 strain expressing only the malonyl‐CoA reductase McrC produced 0.3 and 0.5 g L^−1^ MA, respectively (Figure 1b). This observation suggests that the endogenous succinate‐semialdehyde dehydrogenase (YALI0_F26191g) of *Y. lipolytica* exhibits malonate‐semialdehyde dehydrogenase activity, catalyzing the formation of MA from malonate‐semialdehyde similarly to Uga2 in *S. cerevisiae*.^[^ ^6a^ ^]^ To explore whether it is due to the low levels of malonate‐semialdehyde dehydrogenase in *Y. lipolytica*, the malonate‐semialdehyde dehydrogenase Yne1 from *E. coli* was further overexpressed in YMA‐2 and YMA‐3 strain to obtain the strains YMA‐4 and YMA‐5, respectively. However, the production of MA was increased to only 0.43 and 0.56 g L^−1^ in the YMA‐4 and YMA‐5 strains, respectively (Figure 1b). Compared to the malonyl‐CoA pathway, the lower cytoplasmic oxaloacetic acid might have resulted in the Mdc pathway producing a lower titer of MA.^[^ ^6c^ ^]^ Despite the overexpression of YneI, YMA‐5 strain exhibited only a 20% increase over YMA‐3 strain, suggesting that McrC expression may be a rate‐limiting step, as previously reported.^[^ ^24^ ^]^

Compared to the two malonate‐semialdehyde pathways, malonyl‐CoA pathway demonstrated superior efficacy for MA production. To improve MA production by increasing the copy numbers of the malonyl‐CoA hydrolase ScEhd3**, the ScEHD3** gene was randomly integrated into the genome of Po1f strain, taking advantage of the higher non‐homologous recombination efficiency of the *Y. lipolytica*. After integration, 20 transformants with higher MA production than YMA‐1 were screened out (Figure 1d). In the shake flask fermentation, an initial glucose concentration of 50 g L^−1^ was identified as the most efficient concentration for MA production (Figure S1, Supporting Information). The maximum achieved MA titer was increased to 3.0 g L^−1^, representing a 16‐fold increase compared to MA production in *S. cerevisiae* (Figure 1e). Throughout the fermentation process, MA accumulation led to a gradual decrease in the pH of the fermentation broth. At the end of the fermentation, the pH decreased to a low level which might inhibit the cell growth, resulting in residual glucose remaining in the broth. This strain was subsequently named YMA‐6 and selected for further studies.

### 2.2 Characterizing Efficient Malonyl‐CoA Hydrolases to Facilitate MA Production

**Figure 2:**
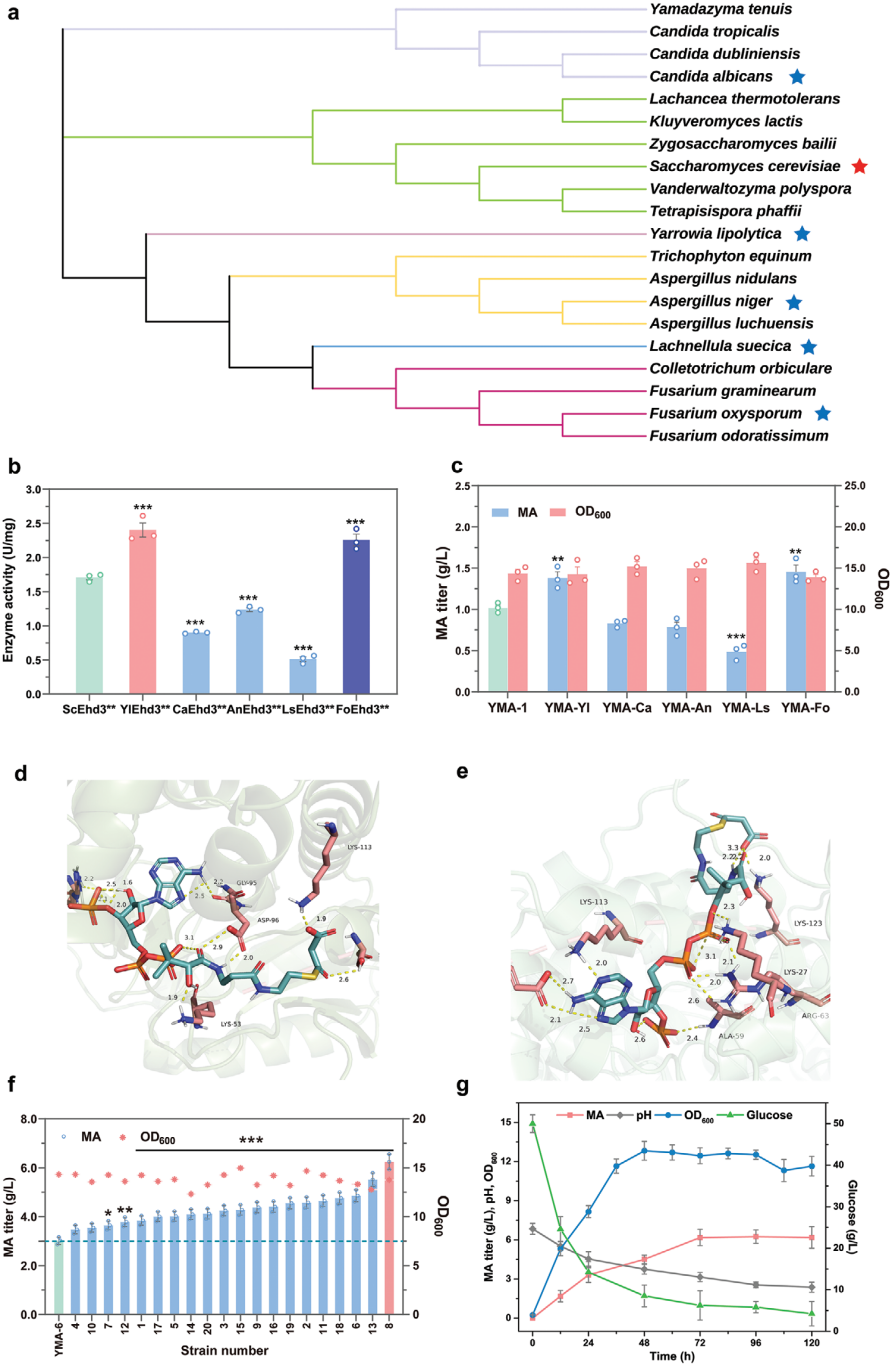
Bioinformatics‐guided screening of efficient malonyl‐CoA hydrolases for MA production. a) Phylogenetic analysis of different Ehd3s. b) Comparison of the enzyme activities of six malonyl‐CoA hydrolases. The asterisks show statistically significant differences from ScEhd3**. c) Comparison of the MA titer produced by six different malonyl‐CoA hydrolases. The asterisks show statistically significant differences from YMA‐1 strain. d,e) Visualization of the best docked model with YlEhd3**, FoEhd3** (receptor) and malonyl‐CoA (ligand), respectively. f) The MA production levels of 20 screened FoEHD3**‐integrated colonies. The asterisks show statistically significant differences from YMA‐6 strain. g) Growth, MA, pH, and glucose consumption of the strain YMA‐7 in shake‐flask fermentation. The data were presented as mean values ± SD from three independent biological replicates (n = 3). The error bars represent the standard deviation (s.d.). Statistical significance was evaluated using one‐way analysis of variance (ANOVA). The asterisks of *, ** and *** denote *p* < 0.05, *p* < 0.01 and *p* < 0.001, respectively.

It has been reported that *Y. lipolytica* exhibits a high flux of malonyl‐CoA.^[^ ^23^ ^]^ However, in addition to being used as a precursor, malonyl‐CoA is integral to numerous cellular metabolic pathways, including lipid and fatty acid synthesis.^[^ ^13^ ^]^ The malonyl‐CoA hydrolase must compete with other metabolic pathways for precursor malonyl‐CoA during the production of MA, making the activity of malonyl‐CoA hydrolase crucial for MA production. To identify more efficient malonyl‐CoA hydrolases for the conversion of malonyl‐CoA to MA, a preliminary screening of fungal malonyl‐CoA hydrolases was performed using a phylogenetic tree based on the amino acid sequence of 3‐hydroxyisobutyryl‐CoA hydrolases (Ehd3s) available in NCBI and UniPort databases. The selection criteria were based on the principle that all Ehd3s possess the conserved amino acids F121 and E124, and exhibit less than 50% amino acid homology with ScEhd3 protein (**Figure** **2**a; Figure S2 Supporting Information). Five different Ehd3s, including YlEhd3 from *Y. lipolytica*, CaEhd3 from *Candida albicans*, AnEhd3 from *Aspergillus niger*, LsEhd3 from *Lachnellula suecica* and FoEhd3 from *F. oxysporum*, were subsequently selected and mutated to evaluate their malonyl‐CoA hydrolase activities and MA‐producing efficiencies.

Initially, five mutated Ehd3 variants, including, YlEhd3** (ScEhd3^F116I‐E119S^), CaEhd3**(CaEhd3^F123I‐E126S^), AnEhd3** (AnEhd3^F118I‐E121S^), LsEhd3** (LsEhd3^F 122I‐E125S^) and FoEhd3** (FoEhd3^F118I‐E121S^), were successfully expressed and purified with a 6×His tag for subsequent enzyme activity assays, using ScEhd3** as a positive control. It was showed that YlEhd3** and FoEhd3** exhibited the relatively highest malonyl‐CoA hydrolase activities, which is 1.4‐fold of ScEhd3**(Figure 2b). Next, to further evaluate the capabilities of these five malonyl‐CoA hydrolases, they were integrated to the genome of Po1f strain with a single‐copy, respectively. Following shake flask fermentation, the strain expressing FoEhd3** produced the highest MA with a titer of 1.45 g L^−1^, while the YlEhd3** integrative strain produced the second highest MA titer of 1.38 g L^−1^, which were both higher than that of YMA‐1 strain using ScEhd3** (Figure 2c). To further analyze the effects of the two malonyl‐CoA hydrolases derived from *Y. lipolytica* and *F. oxysporum*, molecular docking studies were performed to predict the interactions between the two malonyl‐CoA hydrolases (receptor) and malonyl‐CoA (ligand). The optimal docking model was visualized using the PyMOL viewer (Figure 2d,e). It was showed that the affinity of YlEhd3** and malonyl‐CoA was −6.25 kcal/mol and that of FoEhd3** was −5.22 kcal/mol, indicating that the two malonyl‐CoA hydrolases of FoEhd3** and YlEhd3** possess significant potential for MA production.

To further increase MA production, we performed multicopy integration of FoEhd3** or YlEhd3** into the genome of strain YMA‐6, respectively. Consequently, a strain of YMA‐7 was screened out with the highest MA at 6.3 g L^−1^ from the FoEhd3**‐integrated strain (Figure 2f), which was 2.1 times higher than that achieved by YMA‐6 strain. However, no transformants exhibiting significantly higher MA titer than 6.3 g L^−1^ were identified among the YlEhd3**‐integrated strains (Figure S3, Supporting Information). Therefore, YMA‐7 strain was selected for further studies.

### 2.3 Improving the Supplies of Acetyl‐CoA and Malonyl‐CoA to Enhance MA Production

**Figure 3:**
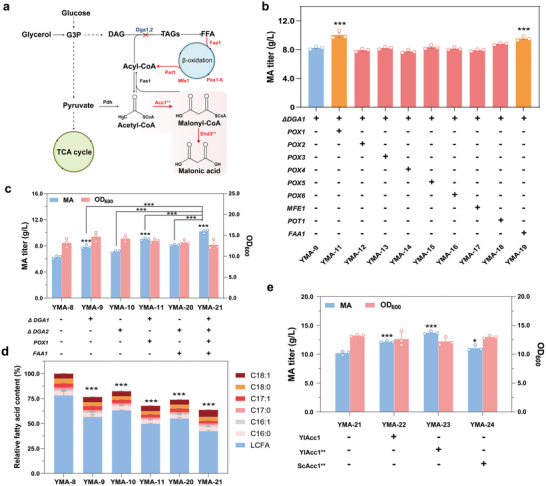
Metabolic engineering for enhancing MA production. a) Schematic diagram of the metabolic engineering strategies for improving MA production. b) Comparison of MA titers in strains with deleted *DGA1* and overexpressed *POX1‐6*, *MFE1*, *POT1* and *FAA1* in YMA‐8 strain. The asterisks show statistically significant differences from YMA‐9 strain. c) The MA titer of the strains with deleted *DGA1* and *DGA2*, and combined with overexpressed *POX1* and/or *FAA1*. d) Comparison of lipids in the indicated engineering strains. The asterisks show statistically significant differences from YMA‐8 strain. e) Comparison of MA titers in engineered YMA‐21 strain with overexpressed YlAcc1, YlAcc1^S667A‐S1178A^ and ScAcc1^S667A‐S1178A^, respectively. The asterisks show statistically significant differences from YMA‐21 strain. The data were presented as mean values ± SD from three independent biological replicates (n = 3). The error bars represent the standard deviation (s.d.). Statistical significance was evaluated using one‐way analysis of variance (ANOVA). The asterisks of *, ** and *** denote *p* < 0.05, *p* < 0.01 and *p* < 0.001, respectively.

To increase the malonyl‐CoA flux for MA production, we first tried to improve the supply of its precursor of acetyl‐CoA by redirecting the carbon metabolic flux from lipid synthesis to acetyl‐CoA by metabolic engineering (**Figure** **3**a). It has been previously found that blocking the flow of triacylglycerol (TAG) precursors by disrupting diacylglycerol (DGA) can increase the supply of acetyl‐CoA in *Y. lipolytica*,^[^ ^14^ ^]^ which is an effective strategy to increase the titer of malonyl‐CoA derivatives. Therefore, the *DGA1* and *DGA2* genes were deleted in strain YMA‐8 to generate strains YMA‐9 and YMA‐10, respectively (Figure 3b,c). As expected, this genetic modification resulted in a 21.5% and 13.9% increase in MA production for strains YMA‐9 and YMA‐10, respectively, without significant changes in the biomass (Figure 3c). In addition, we found that the intracellular lipids produced by YMA‐9 and YMA‐10 strains were reduced by 23.4% and 17.6% compared to the YMA‐8 strain, respectively (Figure 3d). These findings indicated that the deletion of *DGA1* and *DGA2* genes decreased the metabolic flux toward fatty acids synthesis, thus increasing the availability of acetyl‐CoA. This observation might further increase the flux from acetyl‐CoA to malonyl‐CoA, thereby facilitating MA production.

Another effective strategy for enhancing MA production is to promote the degradation of storage fatty acids through upregulating the β‐oxidation pathway to recycle acetyl‐CoA. In *Y. lipolytica*, the initial step of fatty acid β‐oxidation is catalyzed by six distinct acyl‐CoA oxidases (Pox1‐6), while the second and third steps are mediated by a multifunctional enzyme (Mfe1). The final step is catalyzed by 3‐ketoacyl‐CoA‐thiolase (Pot1).^[^ ^25^ ^]^ Overexpression of β‐oxidation‐related genes can accelerate β‐oxidation, thereby increasing the acetyl‐CoA supply.^[^ ^26^ ^]^ In addition, four distinct fatty acid acyl‐CoA synthases are responsible for activating fatty acids of varying chain lengths prior to their entry into peroxisomes in *S.cerevisiae*. Conversely, only one acyl‐CoA synthase (Faa1) has been identified in the cytoplasm in *Y. lipolytica*.^[^ ^27^ ^]^ Thus, we tried to improve the β‐oxidation pathway and the turnover of acyl‐CoA to acetyl‐CoA by overexpressing nine genes, namely *POX1‐6*, *MFE1*, *POT1* and *FAA1* in YMA‐8 strain, respectively. The results showed that the titer of MA was increased by 21.6% and 15.6% in the resulting strain of YMA‐11 and YMA‐21, which overexpressed *POX1* and *FAA1*, respectively (Figure 3b). In contrast, the overexpression of *POT1* has no significant influence on MA production, although it has been reported that its overexpression showed significant effect in other studies.^[^ ^16^, ^17^ ^]^ Combining all the findings, both of *POX1* and *FAA1* genes were overexpressed by integrating them into the *DGA1* and *DGA2* loci, respectively, to create the YMA‐21 strain. The MA production of YMA‐21 strain was increased by 63.3% to 10.2 g L^−1^ compared with that achieved in YMA‐8 strain. Additionally, the intracellular lipids level was significantly reduced by 36.3% (Figure 3d). These findings indicate that this ‘restrain‐pull’ strategy is effective in redirecting the metabolic flux from lipid to MA production.

Although an increase in acetyl‐CoA supply could improve MA production, considering the key enzyme of acetyl‐CoA carboxylase (Acc1) which is responsible for converting acetyl‐CoA to malonyl‐CoA, was directly phosphorylated and inactivated by Snf1 kinase in *S. cerevisiae*.^[^ ^28^ ^]^ and resulted in a deficiency of malonyl‐CoA, we further investigated the role of acetyl‐CoA carboxylase in MA production in *Y. lipolytica*. In *S. cerevisiae*, the two phosphorylation sites (S659 and S1157) of Acc1 were mutated from Ser to Ala to inhibit the phosphorylation by Snf1.^[^ ^29^ ^]^ However, there are fewer studies on the phosphorylation site of Acc1 in *Y. lipolytica*. To address this, we compared the amino acid sequence of YlAcc1 with that of ScAcc1 for homology and found that the amino acids S667 and S1178 are relatively conserved with ScAcc1 (S659 and S1157) (Figure S4, Supporting Information), suggesting that these residues may be the potential phosphorylation sites. To verify our hypothesis, the wild‐type YlAcc1 and its mutant YlAcc1^S667A‐S1178A^ (YlAcc1**) were both integrated into the MA‐engineered YMA‐21 strain to construct YMA‐22 and YMA‐23 strain, respectively. It was found that expressing YlAcc1** in YMA‐23 strain increased the titer of MA by 34.8% to 13.8 g L^−1^ compared with YMA‐21 strain, which was higher than the 12.7 g L^−1^ of YMA‐22 strain expressing the wild‐type YlAcc1. Surprisingly, the expression of ScAcc1^S659A‐S1157A^ (ScAcc1**) mutant did not significantly increase MA production of YMA‐24 strain (Figure 3e). These findings suggested that the YlAcc1 might be also negatively regulated by Snf1 kinase through phosphorylation at S667 and S1178, which was similar to the regulation observed in *S. cerevisiae*.

### 2.4 Optimizing the Culture Conditions to Enhance MA Production

**Figure 4:**
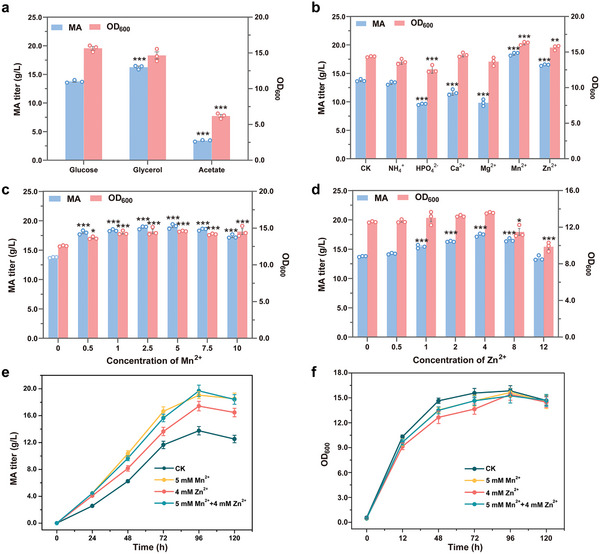
Fermentation optimization of the engineered YMA‐23 strain. a) MA production by YMA‐23 strain with glucose, glycerol, and acetate. The asterisks show statistically significant differences from glucose as carbon source. b) MA production by YMA‐23 strain with different inorganic ions. The asterisks show statistically significant differences from YPG medium. c,d) MA production with variable concentrations of Mn^2+^ and Zn^2+^ by YMA‐23 strain, respectively. The asterisks show statistically significant differences from YPG medium. e, f) Growth of YMA‐23 strain with variable concentrations of Mn^2+^ and Zn^2+^, respectively. The data were presented as mean values ± SD from three independent biological replicates (n = 3). The error bars represent the standard deviation (s.d.). Statistical significance was evaluated using one‐way analysis of variance (ANOVA). The asterisks of *, ** and *** denote *p* < 0.05, *p* < 0.01 and *p* < 0.001, respectively.

In addition to glucose, glycerol is frequently employed as the sole carbon source for *Y. lipolytica*. It was shown that the supplementation of glycerol resulted in an increase in the pools of acetyl‐CoA and G3P, thereby providing sufficient precursors for the MVA pathway and lipid accumulation.^[^ ^30^ ^]^ In addition, acetyl‐CoA can be supplied through acetate, which is an economical substrate. To determine the optimal growth conditions for the engineered strains, we conducted shake flask fermentations utilizing glucose, glycerol, and acetate as carbon sources. The results indicated that glycerol resulted in a higher titer of MA compared to glucose, although it was not supportive of strain growth. Conversely, acetate proved to be detrimental to the growth of the engineering strain, yielding the lowest titer of MA (**Figure** **4**a). Consequently, glycerol was selected as the carbon source for subsequent investigations.

A large number of inorganic ions, including NH_4_^+^, HPO_4_^2−^, Mg^2+^, Ca^2+^, Mn^2+^and Zn^2+^ were found to be essential for microbial growth, metabolic regulation and enzyme activities. In the process of microbial growth and metabolism, NH_4_^+^ could be utilized as a nitrogen source, while phosphorus was an essential element of ATP, ADP, cell membrane, protein and nucleic acid.^[^ ^31^ ^]^ Metal ions can effectively regulate the metabolic pathways and enzyme activities during the fermentation process primarily because of their significant roles as cofactors for a large number of key enzymes.^[^ ^32^ ^]^ To investigate whether these inorganic ions could improve MA production, we supplemented the culture medium with 5 mm NH_4_Cl, 5 mm Na_2_HPO_4_, 0.5 mm MnCl_2_, 8 mm ZnCl_2_, 100 mm MgCl_2_, and 50 mm CaCl_2_ to study their effects on MA production using YMA‐23 strain (Figure 4a).^[^ ^8^ ^]^ After shake flask fermentation, the titers of MA were all lower than that of the control when HPO_4_^2−^, Mg^2+,^ and Ca^2+^ were added to the culture medium. In addition, the MA level was almost the same as that of the control after fermenting with 5 mm NH_4_Cl, indicating that A^+^ had no significant effect on MA production. Surprisingly, the final titers of MA reached to 18.5 and 16.5 g L^−1^ when the YMA‐23 strain were fermented with 0.5 mM MnCl_2_ or 8 mM ZnCl_2_, which was increased by 34.1% and 19.7%, respectively (Figure 4b). These results indicated that Mn^2+^ and Zn^2+^ were favorable to MA production. Next, the concentration of MnCl_2_ and ZnCl_2_ were optimized, revealing that 5 mM MnCl_2_ and 4 mM ZnCl_2_ were most effective for MA production. In detail, the titer of MA increased by 38.4% to 19.1 g L^−1^ with the addition of 5 mM MnCl_2_ and by 26.7% to 17.4 g L^−1^ with the addition of 4 mM ZnCl_2_ to the culture during shake flask fermentation (Figure 4c,d). However, it is noteworthy that the combined addition of 5 mM MnCl_2_ and 4 mM ZnCl_2_ did not result in an additive effect, suggesting the possibility of a shared mechanism of action (Figure 4e,f).

To further investigate the mechanisms by which Mn^2+^and Zn^2+^ improve MA production, we next analyzed the activity of malonyl‐CoA hydrolase in response to different concentrations of Mn^2+^and Zn^2+^. Our findings indicate that the elevated concentrations (>100 µM) of both Mn^2+^and Zn^2+^ inhibited the activity of malonyl‐CoA hydrolase. The optimal concentrations for Mn^2+^and Zn^2+^ were determined to be 10 and 1 µM, respectively. In details, the activity of malonyl‐CoA hydrolase was increased by 24.4% in response to 10 µm Mn^2+^ and by 66.7% in response to 1 µM Zn^2+^ (Figure S5, Supporting Information). This observation might be attributed to the fact that the enzymatic assays were conducted in vitro using the purified malonyl‐CoA hydrolase, which exhibits specific optimal concentrations for Mn^2+^and Zn^2+^. Upon the addition of varying concentrations of MnCl_2_ and ZnCl_2_ to the fermentation culture, the intracellular levels of Mn^2+^and Zn^2+^ were maintained within a specific range,^[^ ^33^ ^]^ subsequently activating malonyl‐CoA hydrolase and promoting the production of MA.

**Figure 5:**
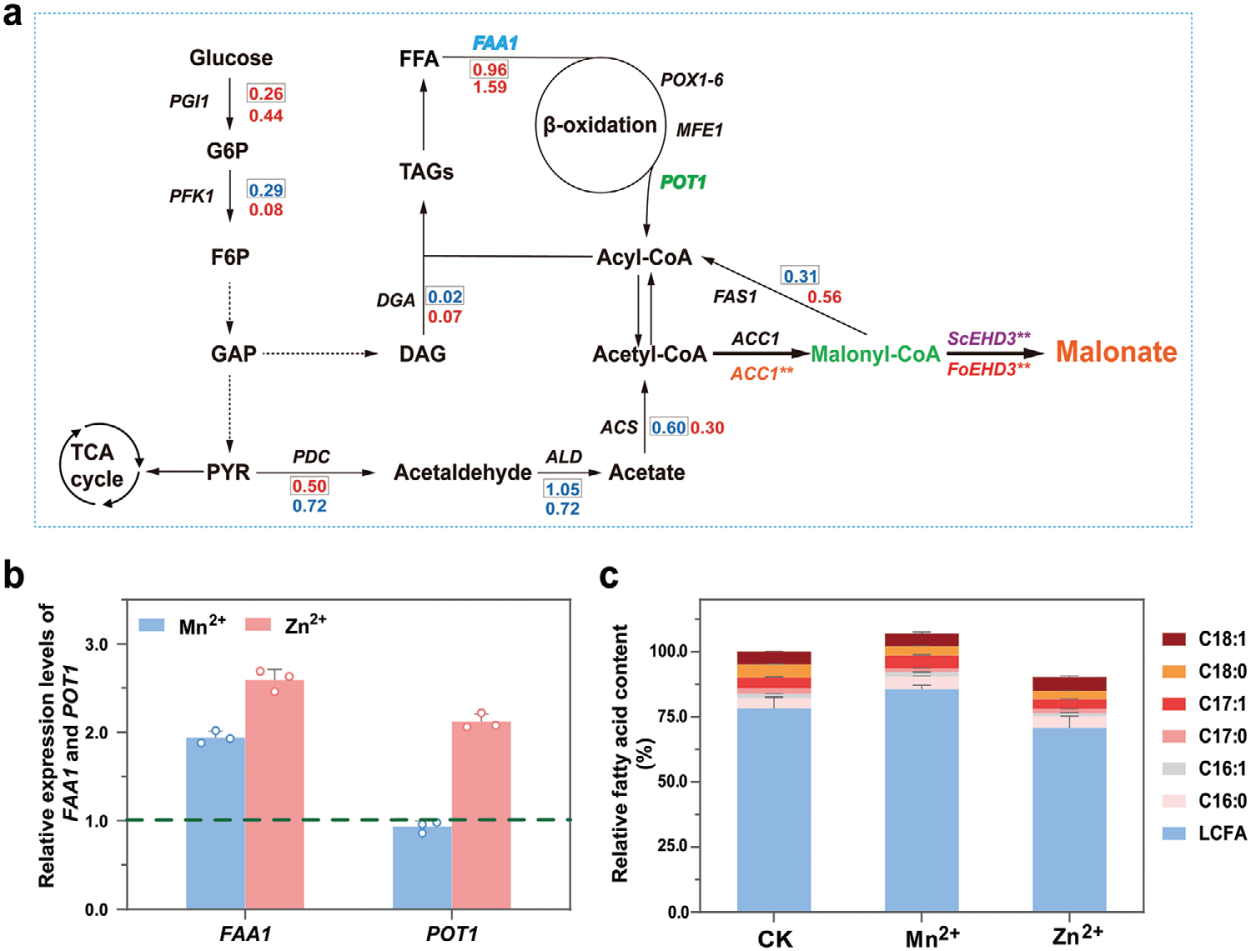
Mechanism analysis of Mn^2+^ and Zn^2+^ ions. a) Transcriptional changes of the genes involved MA synthesis pathway. b) Relative expression levels of *FAA1* and *POT1*. c) Comparison of lipids of YMA‐23 strain in the medium with Mn^2+^ and Zn^2+^. The number indicates the ratio of expression levels (Log_2_FC), red and blue numbers indicate the significantly up‐regulation and down‐regulation levels, respectively. The box‐shaped and none‐box‐shaped numbers indicate the transcriptome results of samples Mn^2+^ and Zn^2+^, respectively. G‐6‐P, Glucose‐6‐phosphate; F‐6‐P, Fructose‐6‐phosphate; GAP, Glyceraldehyde‐3phosphate; PYR, Pyruvate; DAG, Diacylglycerol; TAG, Triacylglycerols; FFA, Free fatty acid. The data were presented as mean values ± SD from three independent biological replicates (n = 3). The error bars represent the standard deviation (s.d.).

We further employed RNA‐sequencing (RNA‐seq) analysis to investigate the ameliorative effects of Mn^2+^ and Zn^2+^ ions on MA production. In comparison to standard fermentation without Mn^2+^ and Zn^2+^ ions, 472 differentially expressed genes (DEGs) were identified in response to 5 mM Mn^2+^, comprising 192 up‐regulated and 280 down‐regulated DEGs (Figure S6a, Supporting Information). While 335 DEGs were identified in response to 4 mm Zn^2+^, including 110 up‐regulated and 225 down‐regulated DEGs (Figure S6b, Supporting Information). Notably, over 80% of the DEGs in response to Mn^2+^ and Zn^2+^ were found to be identical, which elucidates why the combined presence of Mn^2+^ and Zn^2+^ ions did not significantly increase MA titer in shake flask fermentation. Kyoto Encyclopedia of Genes and Genomes (KEGG) enrichment analysis revealed that the most significant DEGs were associated with metabolic pathways, biosynthesis of secondary metabolites, membrane metabolism and carbon metabolism (Figure S6c,d, Supporting Information). These findings suggested that the improved elevated metabolism levels and membrane viability were conducive to increase MA production. Given that the supply of acetyl‐CoA is tightly regulated by lipid metabolism in *Y. lipolytica*, we analyzed the gene expression levels of genes involved in lipid metabolism when the YMA‐23 strain was fermented with either 5 mm Mn^2+^or 4 mm Mn^2+^. Among the genes involved in DGA metabolism and the degradation of storage fatty acids, only the expression levels of *FAA1* and *POT1* were observed to be upregulated in the presence of Mn^2+^ and Zn^2+^ ions (**Figure** **5**a,b), which might potentially facilitate the degradation of fatty acid. Correspondingly, the lipid yield was increased by 7.2% and decreased 10.3% upon the addition of 5 mm Mn^2+^or 4 mm Mn^2+^, respectively, to the culture medium (Figure 5c). These findings support our hypothesis that Mn^2+^ and Zn^2+^ ions can enhance the production of MA by regulation fatty acid metabolism and subsequently increasing the supply of acetyl‐CoA through the upregulation of *FAA1* and *POT1* expression.

### 2.5 MA Fermentation in Scale‐Up Bioreactors

**Figure 6:**
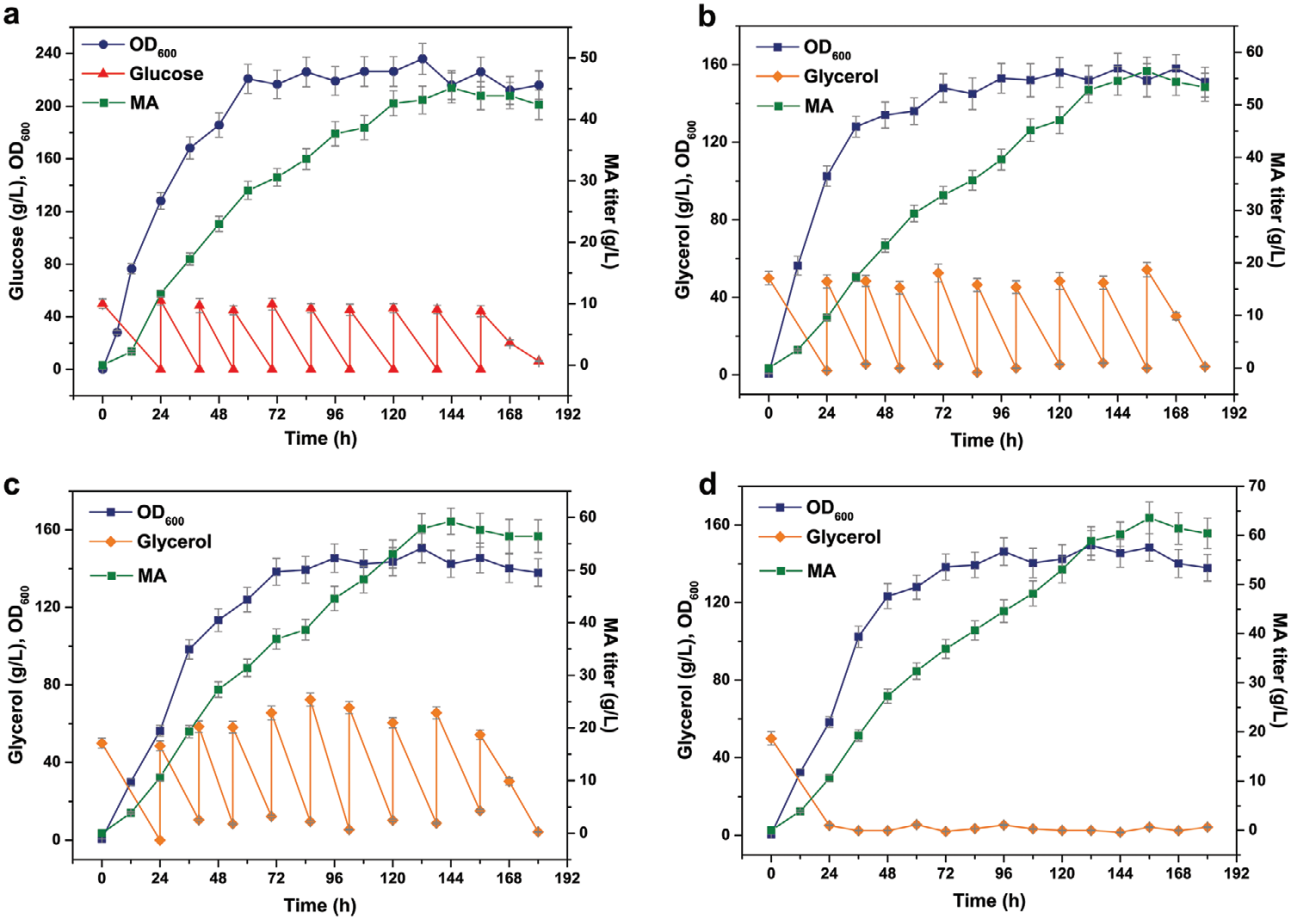
Fed‐batch fermentation of YMA‐23 strain in a 5 L bioreactor. a,b) Growth, glucose or glycerol consumption and MA production of the YMA‐23 strain under the condition of supplementing the glucose or glycerol to a final concentration of 50 g L^−1^ in a single addition once the glucose or glycerol was depleted, respectively. c) Growth, glycerol consumption, and MA production of the YMA‐23 strain under the condition of pulse feeding of glycerol and Mn^2+^. d) Growth, glycerol consumption, and MA production of the YMA‐23 strain under the condition of limiting the glycerol concentration in the fermentation broth to below 5 g L^−1^ with 5 mm Mn^2+^. The values and error bars reflected the mean ± standard deviations (SD) of three biological replicates (n = 3). The error bars represent the standard deviation (s.d.).

To evaluate the potential productivity of MA by YMA‐23 strain and to further enhance MA production, a 5‐L fed‐batch fermentation was performed. Four distinct feeding strategies were designed using glucose or glycerol as the carbon source and maintaining a pH of 6, which proved to be the most suitable for the MA production (Figure S7, Supporting Information). In the first strategy, the initial glucose concentration was set at 50 g L^−1^, and it was supplemented to a final concentration of 50 g L^−1^ in a single addition once the glucose was depleted. The MA titer reached 45.1 g L^−1^ at 144 h, with a productivity of 0.31 g L^−1^ h^−1^ (**Figure** **6**a). This MA production level was more than twofold higher than that achieved in shake flask fermentation. In addition, the engineered YMA‐23 strain exhibited a rapid growth rate, achieving its maximum biomass of 220 (OD_600_) only after 50 h's fermentation. Given that glycerol was found to be more favorable for MA production in shake flask fermentation, it was chosen as the carbon source for the second feeding strategy. The titer of MA reached 54.3 g L^−1^ at 156 h, with a productivity of 0.35 g L^−1^ h^−1^, even though the highest biomass only reached 150 (OD_600_, Figure 6b). Furthermore, in shake flask fermentation, it was found that the addition of 5 mM MnCl_2_ and 4 mM ZnCl_2_ could enhance MA production, with MnCl_2_ showing a particularly significant effect. Then, fed‐batch fermentation was carried out using glycerol as a carbon source with the addition of 5 mM MnCl_2_ to the culture medium. The fed‐batch fermentation results showed that YMA‐23 was able to produce 59.3 g L^−1^ at 156 h with a productivity of 0.38 g L^−1^ h^−1^ (Figure 6c). In the final strategy, following the depletion of glycerol in the initial media, a constant‐rate (0.28 mL/min) feeding strategy was implemented to ensure that the glycerol concentration in the fermentation broth remained below 5 g L^−1^.^[^ ^34^ ^]^ This approach resulted in a production of 63.6 g L^−1^ MA at 156 h by YMA‐23 strain, with an enhanced productivity of 0.41 g L^−1^ h^−1^ (Figure 6d), which is the highest titer of MA reported. The analysis of the fermentation broth revealed that the accumulation of citric acid among the organic acids, achieving concentrations of ≈10 g L^−1^, whereas succinic acid and malic acid were detected at concentrations below 5 g L^−1^ (Figure S8, Supporting Information). These findings suggest that the constant‐rate feeding strategy enhances glycerol utilization while minimizing the synthesis of by‐products in comparison to the fed‐batch feeding approach.

## 3 Discussion

With the ability to grow on various carbon sources including waste cooking oil,^[^ ^35^ ^]^ excellent acid tolerance, and being unaffected by glucose repression,^[^ ^36^ ^]^ *Y. lipolytica* has been recognized as an ideal strain for biosynthesis of high value‐added products based on its abundant acetyl‐CoA and malonyl‐CoA precursors.^[^ ^37^ ^]^ In this study, an effective malonyl‐CoA pathway was constructed to produce MA in *Y. lipolytica* by introducing the malonyl‐CoA hydrolase from *S. cerevisiae* and *F. oxysporum*. Enhancing the fluxes of acetyl‐CoA and malonyl‐CoA enabled the engineered strain to produce a high level of MA both in the shake flasks and the scaled‐up fermentation. Compared with other MA‐producing method, this engineered strain could synthesize MA with higher titer and productivity.

Microbial production of MA has been successfully performed in *E. coli*, *M. thermophila* and *S. cerevisiae* via three intermediates of β‐alanine,^[^ ^6a^ ^]^ malonate‐semialdehyde,^[^ ^6c^ ^]^ and malonyl‐CoA,^[^ ^8^ ^]^ respectively. The β‐alanine pathway requires the involvement of more than six enzymes to convert glucose to MA. This process is further complicated by competition for carbon sources from other metabolic pathways and the low activity of semialdehyde dehydrogenase (YneI), which results in the accumulation of the byproduct of β‐alanine. These factors collectively contribute to a low titer of MA.^[^ ^6a^ ^]^ Our previous study has demonstrated that the β‐alanine pathway is less efficient than the malonyl‐CoA pathway in *S. cerevisiae* for MA production.^[^ ^7^, ^8^ ^]^ However, the cytoplasmic levels of malonyl‐CoA *S. cerevisiae* might insufficient for optimal MA production. Consequently, *Y. lipolytica* might serve as a more suitable host for MA production via malonyl‐CoA pathway. Currently, no studies have reported the MA production by the two malonate‐semialdehyde pathways in yeast, the Mdc pathway and the McrC pathway. The Mdc pathway is shorter than the β‐alanine pathway, however, the poor acid tolerance of *M. thermophila* limits its utility as a host for synthesizing MA through MDC pathway.^[^ ^6c^ ^]^ Conversely, the Mdc pathway may perform better in *Y. lipolytica* due to its excellent acid tolerance. In addition, the McrC pathway, another malonate‐semialdehyde pathway, has significant advantages in the production of 3‐HP.^[^ ^22^, ^24^ ^]^ To develop an effective synthesis pathway for MA, we evaluated the efficiencies of the malonyl‐CoA pathway and two malonate‐semialdehyde pathways of Mdc pathway, and McrC pathway for MA production in *Y. lipolytica*. The malonyl‐CoA pathway was constructed by introducing malonyl‐CoA hydrolase derived from the mutated 3‐hydroxyisobutyryl‐CoA hydrolase ScEhd3** for producing MA in *S. cerevisiae* in our previously study, the highest titer of MA was only 13.6 mg L^−1^ after shake flask fermentation before engineering the MA synthetic pathway.^[^ ^8^ ^]^ By expressing the malonyl‐CoA hydrolase from ScEhd3** in the *Y. lipolytica* Po1f strain, the engineered YMA‐1 strain produced 1.0 g L^−1^ MA (Figure 1b), which was a 73.5‐fold increase compared to that produced by the same malonyl‐CoA pathway in *S. cerevisiae*. This titer was also higher than those produced by the two malonate‐semialdehyde pathways, namely the Mdc pathway and McrC pathway. Future studies aimed to increase MA production via malonate‐semialdehyde pathway should focus on identifying more efficient enzymes, specifically the oxaloacetate decarboxylase, malonyl‐CoA reductase and malonate‐semialdehyde dehydrogenase. These findings indicated that the enrichment of MA synthesis precursors, especially acetyl‐CoA and malonyl‐CoA in *Y. lipolytica*, making this non‐traditional yeast an ideal host for MA production via the malonyl‐CoA pathway.

At present, only the mutation of 3‐hydroxyisobutyryl‐CoA hydrolase (ScEhd3**) in *S. cerevisiae* has been reported to have the malonyl‐CoA hydrolase activity. In this study, the malonyl‐CoA pathway was constructed by overexpressing the malonyl‐CoA hydrolase ScEhd3** in *Y. lipolytica*, resulting in a production yield of 3.0 g L^−1^ following shake flask fermentation (Figure 1e). However, the conversion rate was still very low, given the utilization of 50 g L^−1^ glucose. These findings indicated that the activity of malonyl‐CoA hydrolysis is crucial for efficient MA production. To identify more efficient malonyl‐CoA hydrolases for the conversion of malonyl‐CoA to MA, a preliminary screening for malonyl‐CoA hydrolases from fungal origin was performed using a phylogenetic tree based on the amino acids of 3‐hydroxyisobutyryl‐CoA hydrolase ScEhd3 and its conserved amino acids for malonyl‐CoA hydrolase (F121I and E124S) available in NCBI and UniPort databases. Two new malonyl‐CoA hydrolases from *Y. lipolytica* and *F. oxysporum* with higher malonyl‐CoA hydrolase activities than ScEhd3** were identified. The MA titer was increased to 6.3 g L^−1^ by overexpressing FoEhd3** (Figure 2g). To increase the production of MA, further study might be needed to explore more efficient malonyl‐CoA hydrolases from other origins including plants, animals as well as bacteria.

In addition to malonyl‐CoA hydrolase activity, the malonyl‐CoA supply is another limiting step for MA production, the intracellular levels of which is tightly regulated by the accumulation of its precursor of acetyl‐CoA. In *Y. lipolytica*, more acetyl‐CoA and malonyl‐CoA are used for lipid synthesis, as wildtype strains can accumulate lipids up to 70% of dry biomass.^[^ ^38^ ^]^ We employ a ‘restrain–pull’ strategy effectively switching the carbon metabolic flux from lipid to acetyl‐CoA by deleting two diacylglycerol acyltransferases of Dga1 and Dga2 to decrease the consumption of acetyl‐CoA, overexpressing the enzymes involved in β‐oxidation pathway and acyl‐CoA synthase Faa1 to increase the acetyl‐CoA supply. The titer of MA was significantly increased to 10.2 g L^−1^ through reducing the synthesis of lipids (Figure 3c,d). The regulatory mechanism of the acetyl‐CoA carboxylase (Acc1) in *Y. lipolytica* has not been well studied yet, although it has been reported that the activity of ScAcc1 could be tightly regulated by Snf1 kinase by direct phosphorylation.^[^ ^28^ ^]^ Here, by comparing the amino acid sequence of ScAcc1 and YlAcc1, we found that YlAcc1 also shared the predicted phosphorylation sites (S667 and S1178) of Snf1 kinase. The MA production was increased 34.8% by overexpressing the mutated YlAcc1^S667A‐S1178A^ (Figure 3e), indicating that similar regulation mechanism might be exited for YlAcc1 as the ScAcc1 by protein kinase of Snf1.

In fed‐batch fermentation, despite the improved MA level using glycerol as a carbon source, the highest biomass of YMA‐23 reached only ≈ 150 (OD_600_), which was just 68.2% of that using glucose as a carbon source (Figure 5a,b). The addition of glycerol resulted in the elevation of the acetyl‐CoA pools, facilitating the flux of carbon metabolism to MA thereby competing for some carbon sources, which may have resulted in the low biomass.^[^ ^30b^ ^]^ Owing to the Crabtree effect, *S. cerevisiae* produces a large amount of ethanol in the presence of oxygen and excess glucose, leading to a loss of carbon for the biosynthesis of non‐ethanol chemicals.^[^ ^39^ ^]^ In contrast, *Y. lipolytica* is not inhibited by high sugar concentrations because of its Crabtree‐negative characteristic, and its rate of sugar consumption is much higher than that of other microorganisms.^[^ ^35a^ ^]^ Therefore, the initial concentrations of the carbon sources were usually set to a relatively high level.^[^ ^40^ ^]^ Currently, microbial cell factories utilizing *Y. lipolytica* as host cells exhibit generally low product conversion rates. A significant proportion of carbon sources is diverted toward lipid synthesis and other pathways, resulting in wasted carbon sources,^[^ ^41^ ^]^ which limits the industrial application of *Y. lipolytica* as a host strain. To maximize the utilization of different carbon sources, enhancing the activity of key enzymes within the synthetic pathway emerges as the most effective strategy. Given that lipids constitute the primary by‐products of *Y. lipolytica*, promoting lipid degradation and facilitating their conversion into desired products is also a viable approach. Furthermore, coupling the TCA cycle with the crucial intermediate acetyl coenzyme A via the citric acid cycle can further enhance carbon source utilization. Organelle engineering holds the potential to localize the production of target compounds, thereby minimizing substrate loss and improving the carbon conversion rate.^[^ ^16^, ^42^ ^]^ Additionally, transporter engineering can address the limiting factors such as product inhibition, thereby enhancing overall process efficiency.^[^ ^43^ ^]^

In conclusion, an efficient MA‐producing strain was constructed by the selection of different MA synthetic pathways and identification of the effective malonyl‐CoA hydrolases in this study. To improve the supply of acetyl‐CoA and malonyl‐CoA, the strain was metabolically engineered to improve carbon metabolic flux from lipid to acetyl‐CoA by blocking the flow of TAG, enhancing the β‐oxidation pathway as well as eliminating the inhibition of Acc1 via mutation of its two possible phosphorylation sites. Finally, the titer of MA was increased to 63.6 g L^−1^ by using glycerol as carbon source and supplementing MnCl_2_ in the culture medium in fed‐batch fermentation. This work will facilitate the realization of the industrial production of MA and provide a reference for the production of other malonyl‐CoA derivatives in the *Y. lipolytica*.

## 4 Experimental Section

### Strains, Plasmids, and Culture Medium

*E. coli* DH5α was employed for the construction and propagation of plasmids. The bacterial strains were cultured at 37°C with agitation at 250 rpm in Luria–Bertani (LB) liquid medium (10 g L^−1^ NaCl, 10 g L^−1^ Tryptone, and 5 g L^−1^ Yeast extract) or incubated at 37°C on LB agar plates. When necessary, ampicillin was incorporated into the medium at a final concentration of 100 mg L^−1^. The wild‐type *Y. lipolytica* Po1f strain, sourced from the ARS Culture Collection (NRRL), was selected as the foundational strain for further construction. *Y. lipolytica* strains were cultured in yeast extract peptone dextrose (YPD) liquid medium (10 g L^−1^ Yeast extract, 20 g L^−1^ Tryptone and 20 g L^−1^ Glucose), synthetic dropout (SD) medium (1.7 g L^−1^ yeast nitrogen base, 5 g L^−1^ ammonium sulfate, and 20 g L^−1^ glucose) or incubated at 30°C on plates supplemented with 2 g L^−1^ of agar. Detailed descriptions of all recombinant plasmids and strains employed in this study are provided in Table S1 (Supporting Information).

### DNA Manipulation

Six different EHD3**s including ScEHD3** from S. cerevisiae, YlEHD3** from *Y. lipolytica*, CaEHD3** from C. albicans, AnEHD3** from A. niger, LsEHD3** from *L. suecica* and FoEHD3** from F. oxysporum, the oxaloacetate decarboxylase gene MDC from *O. parapolymorpha*, the malonate‐semialdehyde dehydrogenase gene Yne1 from E. coli, the malonyl‐CoA reductase gene McrC from *C. aurantiacus*, as well as the acetyl‐CoA carboxylase gene ACC1^S659A‐S1157A^ from S. cerevisiae were first codon‐optimized and synthesized by Genewiz (Suzhou, China). Other native promoters, genes, and terminators were amplified from the genomic DNA of Po1f strain. The detailed DNA sequence information of EHD3** genes used in this study was listed in Table S2 (Supporting Information). The EHD3** genes were first cloned into the single‐copy number integrated plasmids pINA1269 and expressed using the hp4d promoter and XPR2 terminator. The plasmids were then linearized and integrated into the genome of Po1f strain. In *Y. lipolytica*, non‐homologous end‐joining (NHEJ) was dominant over homologous recombination (HR).^[^ ^44^ ^]^ To construct YMA‐6 strain, ScEHD3** gene was first amplified and cloned into of pKi‐1 plasmid. The resulting plasmid was used as a template for PCR to obtain the integration fragment of P_ut8_‐ScEHD3**‐T_CYC1_‐LEU2, which was then introduced into Po1f strain and randomly integrated into the genome by endogenous NHEJ repair.^[^ ^45^ ^]^ YMA‐6 strain was subsequently obtained through screening. Following this, the FoEHD3** gene was amplified and assembled with restriction enzymes‐digested expression vectors pKi‐2. The integration fragments P_TEFin_‐FoEHD3**‐T_CYC1_‐URA3 was assembled by fusion PCR and then randomly integrated into the YMA‐6 genome. YMA‐7 strain was also obtained by screening.

Efforts to improve gene targeting efficiency have focused on abolishing the NHEJ pathway by disrupting either ku70.^[^ ^46^ ^]^ To achieve rational metabolic modification of the engineered YMA‐8 strain, the Ku70 gene was deleted in YMA‐7 strain. Genes of *POX1*, *POX2*, *POX3*, *POX4*, *POX5*, *POX6*, *MFE1*, *POT1*, *FAA1*, and *ACC1* were amplified and subsequently assembled with restriction enzyme‐digested expression vectors pKi‐2. The integration fragments were then amplified via PCR and transformed into the related host strain. Following this, the Cre protein was transiently expressed using plasmid pYLXP1, which facilitated the removal of the screening tag.^[^ ^47^ ^]^ The primers used in this study is listed in Table S3 (Supporting Information).

### Screening and Structural Analysis of Malonyl‐CoA Hydrolases

The amino acid sequences annotated as 3‐hydroxyisobutyryl‐CoA hydrolase were acquired from NCBI or UniProt databases and subsequently aligned using ClustalW. The maximum likelihood phylogenetic trees were then generated using MEGA‐11 software based on the LG model,^[^ ^48^ ^]^ with the strength of the nodes determined with 100 bootstrap replicates. Molecular docking of Ehd3** (S/H) (receptor) and malonyl‐CoA (ligand) was performed using the default AutoDock vina setting to calculate the interaction between mutant and malonyl‐CoA and the docking model was visualized using PyMOL.^[^ ^49^ ^]^

### RNA‐seq Analysis

To quantify the relative expression levels of the indicated genes, yeast cells were first cultivated overnight in YPD medium, and cultures (2%) were then inoculated into 50 mL of fresh YPD medium and cultured to an OD_600_ of 0.6–0.8. The cells were then inoculated with or without (as the control group) 5 mm MnCl_2_ or 4 mm ZnCl_2_ for an additional 24 h. Cells were harvested and the total RNA was extracted using hot phenol method.^[^ ^50^ ^]^ Transcriptome sequencing analysis was performed by Vazyme Biotech Co., Ltd. The identification of gene expression differences between the different samples was based on |log2Ratio| ≥ 1 and *q*‐value ≤ 0.05 as the standard. Functional annotation of the genes with significant differential expression was carried out using the Kyoto Encyclo‐pedia of Genes and Genomes (KEGG) database. The pathway enrichment analysis tool OmicShare was used to classify genes at the level of KEGG_B_class (https://www.omicshare.com/tools/Home/Soft /pathwaygsea).^[^ ^51^ ^]^

### Shake Flask Fermentations

Single colonies of recombinant strains were inoculated into 5 mL YPD media, and cultivated overnight (16–18 h) at 30°C and 250 rpm. The culture was then transferred to a 250 mL shake flask containing 50 mL Y_10_P_20_D_50_ (10 g L^−1^ Yeast extract, 20 g L^−1^ Tryptone and 50 g L^−1^ Glucose) with an initial OD_600_ = 0.4, and cultivated at 30°C with shaking at 250 rpm for 3∼5 days. Glycerol at a concentration of 50 g L^−1^ or 50 g L^−1^ sodium acetate was utilized as a substitute for glucose in YPD to optimize the carbon source, respectively. The culture medium was supplemented with 5 mm NH_4_Cl, 5 mm Na_2_HPO_4_, 0.5 mm MnCl_2_, 8 mm ZnCl_2_, 100 mm MgCl_2_, and 50 mm CaCl_2_ to study their effects on MA production. The culture medium was further optimized by the addition of Mn^2+^ ion at concentrations of 0.5, 1.0, 2.5, 7.5, and 10 mm, and Zn^2+^ ion at concentrations of 0.5, 1.0, 2.0, 4.0, 8.0, and 12.0 mm, respectively.

### Enzyme Activity Assay

The six linearized plasmids of p1269‐ScEHD3**‐6×his, p1269‐YlEHD3**‐6×his, p1269‐CaEHD3**‐6×his, p1269‐AnEHD3**‐6×his, p1269‐LsEHD3**‐6×his and p1269‐FoEHD3**‐6×his were first transformed into Po1f strain, respectively. Cells were first cultivated overnight at 30°C in 20 mL of SD‐LEU medium, which were then transferred into 200 mL of SD‐LEU medium and cultivated for an additional 12 h. The six different Ehd3** proteins were purified and used for analyzing the malonyl‐CoA hydrolase activity using 5,5ʹ‐dithiobis‐(2‐nitrobenzoic acid) (DTNB) by the method described in our previous study.^[^ ^8^, ^52^ ^]^

### Quantification of Metabolite in Media

Fermentation samples were centrifuged at 10,000 rpm, and the supernatant was collected for analysis via high‐performance liquid chromatography (HPLC) or liquid chromatography–mass spectrometry (LC‐MS). For HPLC analysis, the supernatant was filtered through a 0.22 µm membrane and analyzed using an Agilent Technologies 1260 Infinity II HPLC system, equipped with an HPX‐87H ion‐exclusion column (300 mm × 7.8 mm; BioRad, CA). The mobile phase consisted of 5 mm H_2_SO_4_ with a flow rate at 0.6 mL min^−1^, and the column temperature was maintained at 50°C. Compounds were detected from 20 µL injections using a refractive index detector. LC‐MS was analyzed using QTRAP® 5500 LC/MS system, equipped with an HSS T3 column (1.8 µm, 2.1 × 100 mm, Waters, CA). The mobile phase consisted of ammonium acetate and Acetonitrile with a flow rate at 0.25 mL min^−1^, and the column temperature was maintained at 40°C. Compounds were detected from 2 µL injections.

### Lipid Extraction and Quantification

The lipids synthesized by *Y. lipolytica*, including palmitate (C16:0), palmitoleate (C16:1), stearate (C18:0), oleate (C18:1), linoleate (C18:2) and long‐chain fatty acid (LCFA), were quantified using a Gas Chromatography coupled to a Flame Ionization Detector (GC‐FID). Samples of 1 mL cell culture were centrifuged at 16,000 rpm for 10 min, after which the supernatant was discarded. Subsequently, 0.5 mL of a 0.5 m sodium hydroxide‐methanol solution and 100 µL methyl tridecanoate (Sigma‐Aldrich) as internal standards. The samples were vortexed for 1 h to ensure the transesterification of lipids into fatty acid methyl esters (FAMEs). The FAMEs were extracted by adding hexane after neutralization with 98% sulfuric acid.^[^ ^53^ ^]^ The separation of the FAME species was performed using an Agilent HP‐INNOWax capillary column. The injection volume was set to 1 µL with a split ratio of 10, and the injection temperature was maintained at 260°C. The column temperature was held constant at 200°C, and helium was used as the carrier gas at a flow rate of 1.5 mL min^−1^. The flame ionization detector (FID) was set at a temperature of 260°C, with helium make up gas, hydrogen, and air flow rates at 25, 30, and 300 mL min^−1^, respectively.^[^ ^54^ ^]^

### Bioreactor Fermentations

Fed‐batch fermentations were performed in a 5 L bioreactor (New Brunswick Bioflo115 system). The initial fermentation was completed with 2 L medium containing 50 g L^−1^ glucose or 50 g L^−1^ glycerol, 20 g L^−1^ peptone, and 10 g L^−1^ yeast extract. The temperature was maintained at 30°C, while the dissolved oxygen was controlled at 35% using an agitation cascade ranging from 200 to 600 rpm. Air was sparged into the fermenter at 4 L min^−1^. The pH was maintained by feeding the ammonia solution, while the foam was prevented by the addition of antifoam 204 (Sigma‐Aldrich). Samples were taken every 12 h to measure OD_600_, glucose concentration, and MA titer. Four distinct feeding strategies were employed for fed‐batch fermentation. In the initial strategy, glucose was utilized as the carbon source and was supplemented to reach a final concentration of 50 g L^−1^ in a single addition once depletion occurred, using YPD medium composed of 10 g L^−1^ yeast extract, 20 g L^−1^ peptone, and 500 g L^−1^ glucose. The second and third strategies involved the use of glycerol as the carbon source, with or without the addition of 5 mM MnCl_2_, respectively. Glycerol was supplemented to a final concentration of 50 g L^−1^ in a single addition upon depletion, using YPG medium consisting of 10 g L^−1^ yeast extract, 20 g L^−1^ peptone, and 500 g L^−1^ glycerol. In the final strategy, after the depletion of glycerol in the initial medium, a constant‐rate feeding approach (0.28 mL min^−1^) was employed to maintain the glycerol concentration in the fermentation broth below 5 g L^−1^.^[^ ^34^ ^]^

### Statistical Analysis

All data were presented as mean values ± SD from three independent biological replicates (n = 3). The significant differences were analyzed by GraphPad Prism 9.0 Project software using one‐way analysis of variance (ANOVA). A *p*‐value of <0.05 was considered statistically significant.

## Conflict of Interest

The authors declare no competing interest.

## Author Contributions

Q.Y. and Y.Z. designed the experiments. Q.Y. performed the experiments. Q.Y. and Y.Z. analyzed the results. Q.Y. and Y.Z. prepared and revised the manuscript. M.T. performed experiments for plasmid construction. Q.Y., Y.Z., M.T., P.D., and Y.D. provided overall guidance on the project and participated in revising the manuscript. Y.D. conceived and directed the project. All authors have given approval to the final version of the manuscript.

## Supporting information



Supporting Information

## Data Availability

The data that support the findings of this study are available from the corresponding author upon reasonable request.
