# Supplementary material for: Engineering Yarrowia lipolytica to Enhance the Production of Malonic Acid via Malonyl‐CoA Pathway at High Titer
Source: Adv Sci (Weinh). 2025 Feb 7;12(12):2411665. doi: 10.1002/advs.202411665 (PMC11947988; doi:10.1002/advs.202411665)
Supplement: Supplementary file 1 — Supporting Information [file ADVS-12-2411665-s001.doc]

**Supporting Information**

**Engineering *Yarrowia lipolytica* to Enhance the Production of Malonic acid via Malonyl-CoA Pathway at High Titer**

Qun Yang ^1,2^, Mengzhen Tian^1^, Ping Dong^1^, Yunying Zhao^1,2,3*^ & Yu Deng^1,2,3*^

^1^School of Biotechnology and Key Laboratory of Industrial Biotechnology of Ministry of Education, Jiangnan University, 1800 Lihu Road, Wuxi, Jiangsu 214122, China

^2^National Engineering Research Center of Cereal Fermentation and Food Biomanufacturing, Jiangnan University, 1800 Lihu Road, Wuxi, Jiangsu 214122, China

^3^Jiangsu Provincial Research Center for Bioactive Product Processing Technology, Jiangnan University, 1800 Lihu Road, Wuxi, Jiangsu 214122, China.

^*^ To whom correspondence should be addressed.

Yu Deng: School of Biotechnology and Key Laboratory of Industrial Biotechnology of Ministry of Education, Jiangnan University, 1800 Lihu Road, Wuxi, Jiangsu 214122, China E-mail: dengyu@jiangnan.edu.cn (Y. Deng);

Yunying Zhao: School of Biotechnology and Key Laboratory of Industrial Biotechnology of Ministry of Education, Jiangnan University, 1800 Lihu Road, Wuxi, Jiangsu 214122, China

E-mail: yunyingzhao@jiangnan.edu.cn (Y. Zhao)

Phone: +86-510-85329031, Fax: +86-510-85918312


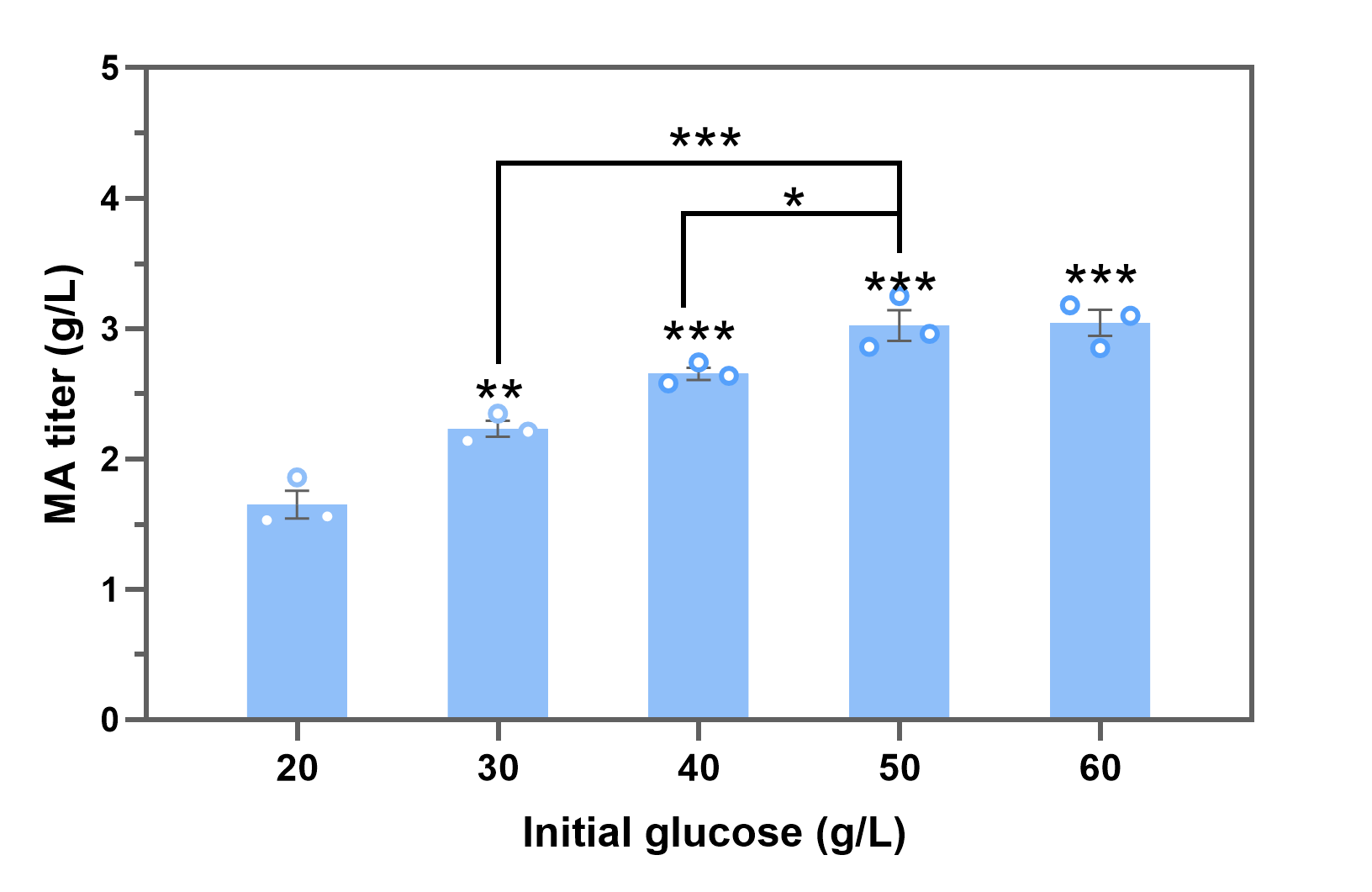


**Figure S1.** The effect of different initial glucose concentrations on the MA production by YMA-6 strain. The data were presented as mean values ± SD from three independent biological replicates (n = 3). The error bars represent the standard deviation (s.d.). Statistical significance was evaluated using one-way analysis of variance (ANOVA). The asterisks of *, ** and *** denote *p* < 0.05, *p* < 0.01 and *p* < 0.001, respectively.

##
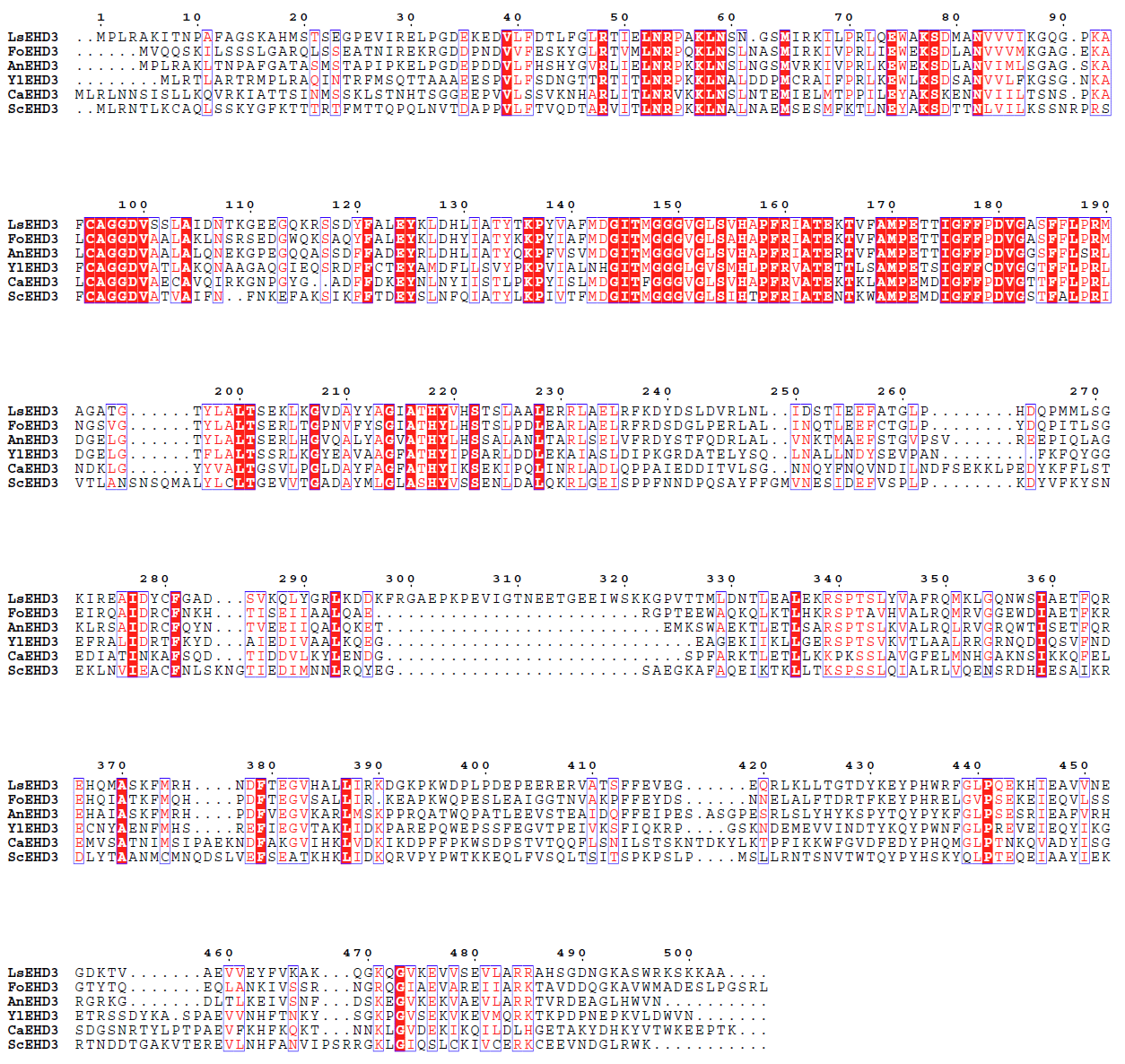


**Figure S2.** Amino acid sequences alignment of six Ehd3s. Sequence alignment of Ehd3 from *S. cerevisiae* (ScEhd3), *Y. lipolytica* (YlEhd3), *C. albicans* (CaEhd3), *A. niger* (AnEhd3), *L. suecica* (CaEhd3) and *F. oxysporum* (FoEhd3). The alignment was performed using ClusterW and output was generated by ESPript3.0. The green box indicates the conserved sequence of the active site.


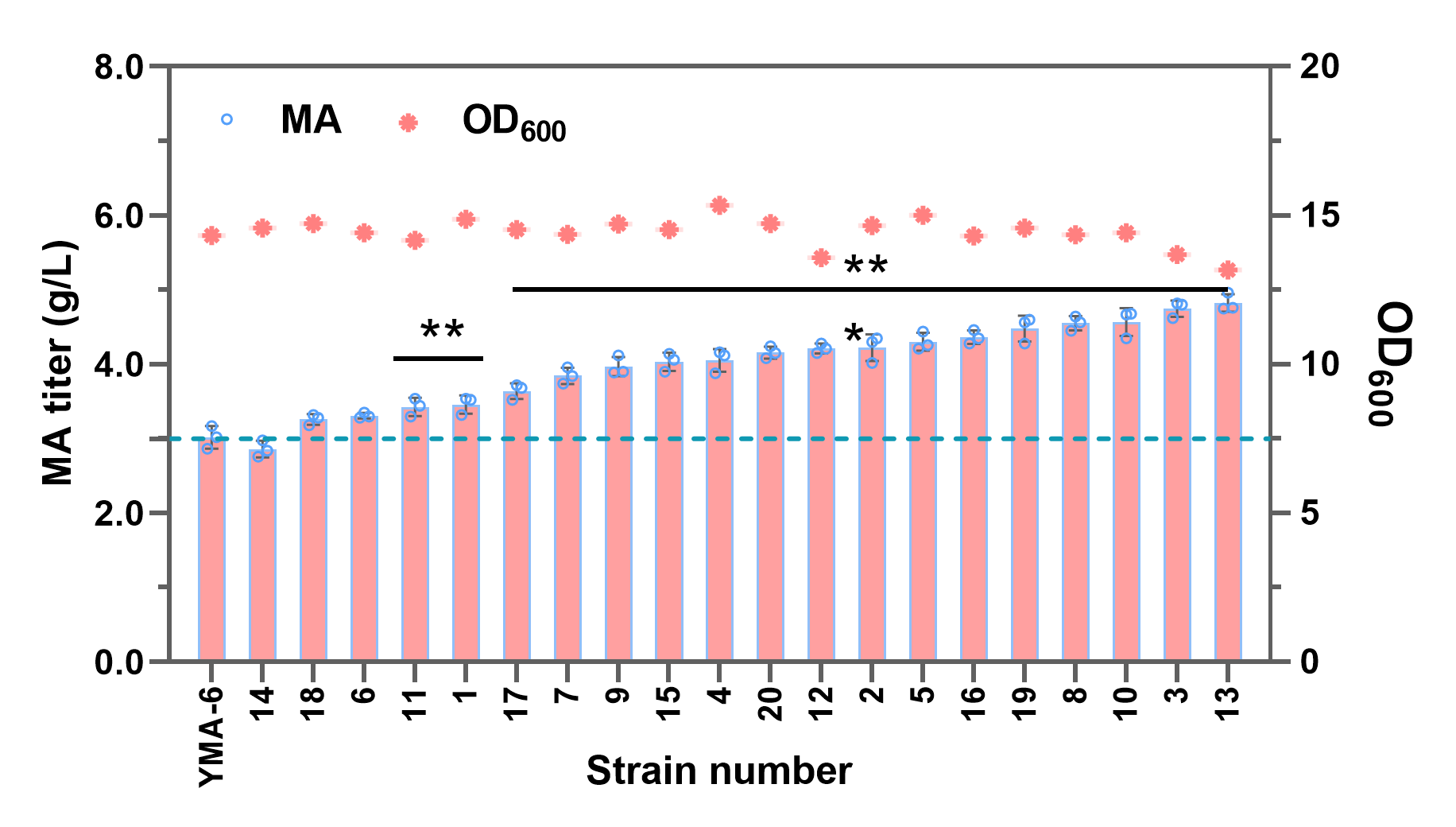


**Figure S3.** The MA production levels of 20 screened *YlEHD3***-integrated colonies. The asterisks show statistically significant differences from YMA-6 strain. The data were presented as mean values ± SD from three independent biological replicates (n = 3). The error bars represent the standard deviation (s.d.). Statistical significance was evaluated using one-way analysis of variance (ANOVA). The asterisks of *, ** and *** denote *p* < 0.05, *p* < 0.01 and *p* < 0.001, respectively.


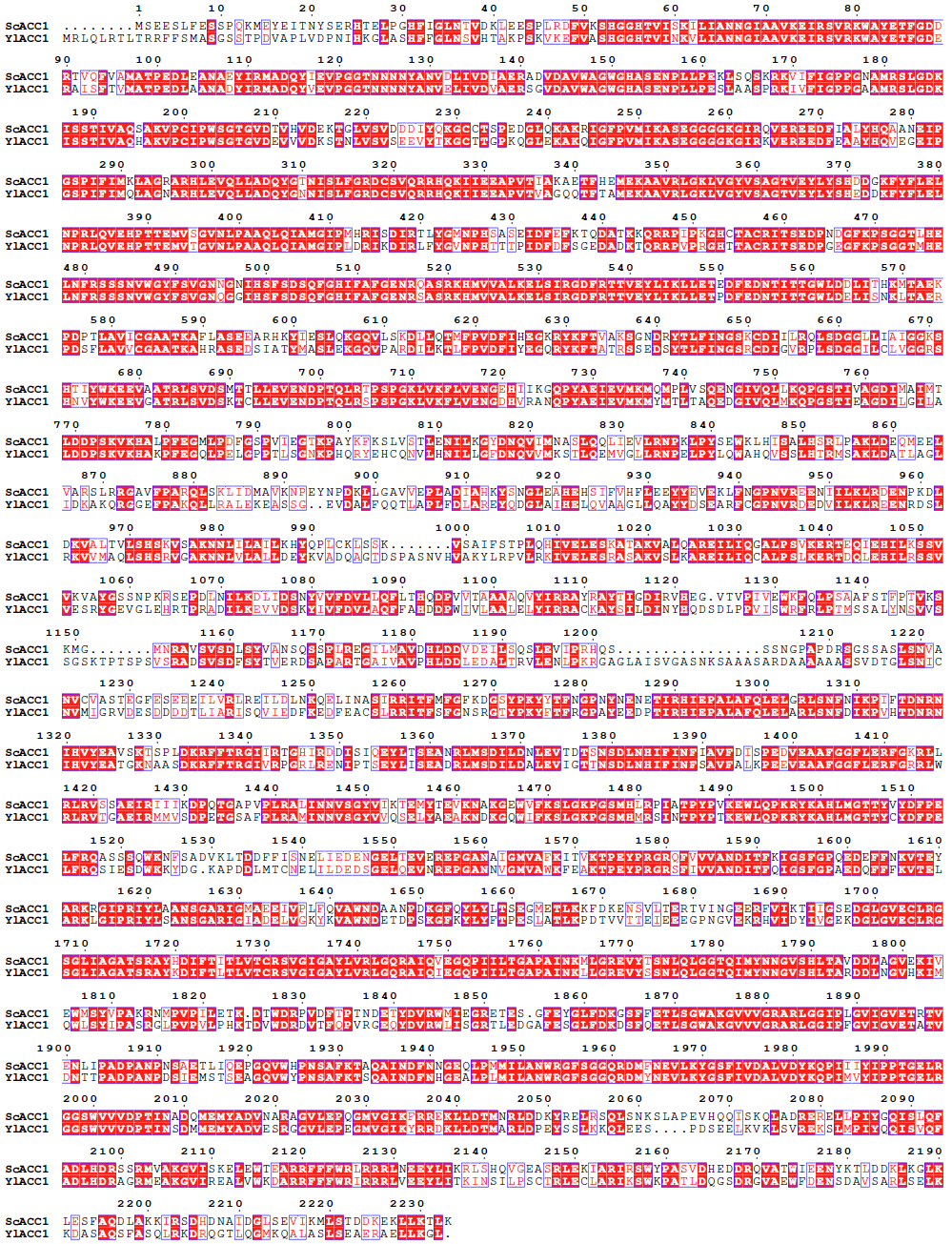


**Figure S4.** Alignment of amino acid sequences of ScAcc1 and YlAcc1. The alignment was performed using ClusterW and output was generated by ESPript3.0. The green box indicates the conserved phosphorylation mutation site.


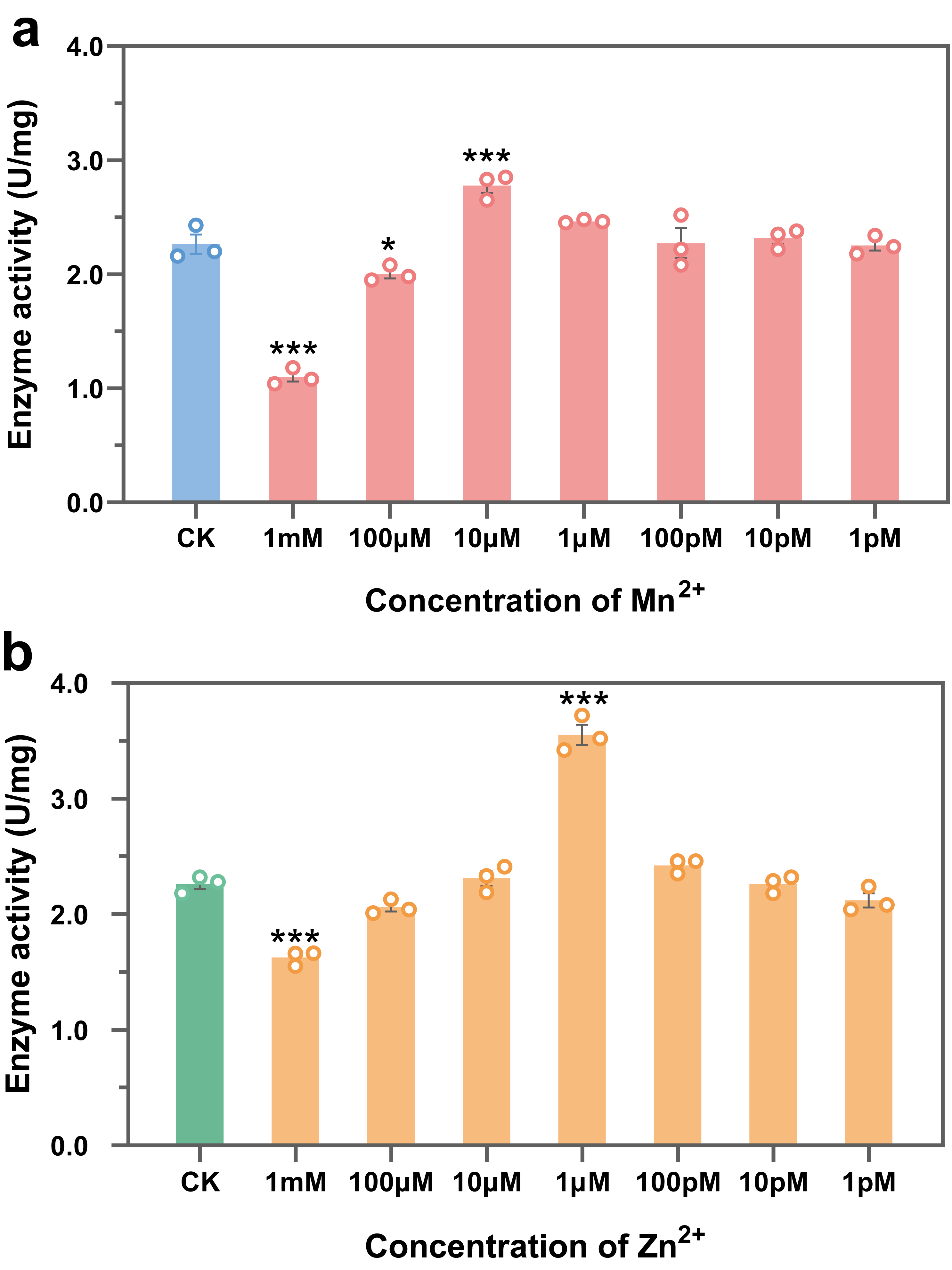


**Figure S5.** Malonyl-CoA hydrolase analysis with variable concentrations of Mn^2+^ (a) and Zn^2+^ (b) ions. The data were presented as mean values ± SD from three independent biological replicates (n = 3). The error bars represent the standard deviation (s.d.). Statistical significance was evaluated using one-way analysis of variance (ANOVA). The asterisks of *, ** and *** denote *p* < 0.05, *p* < 0.01 and *p* < 0.001, respectively.


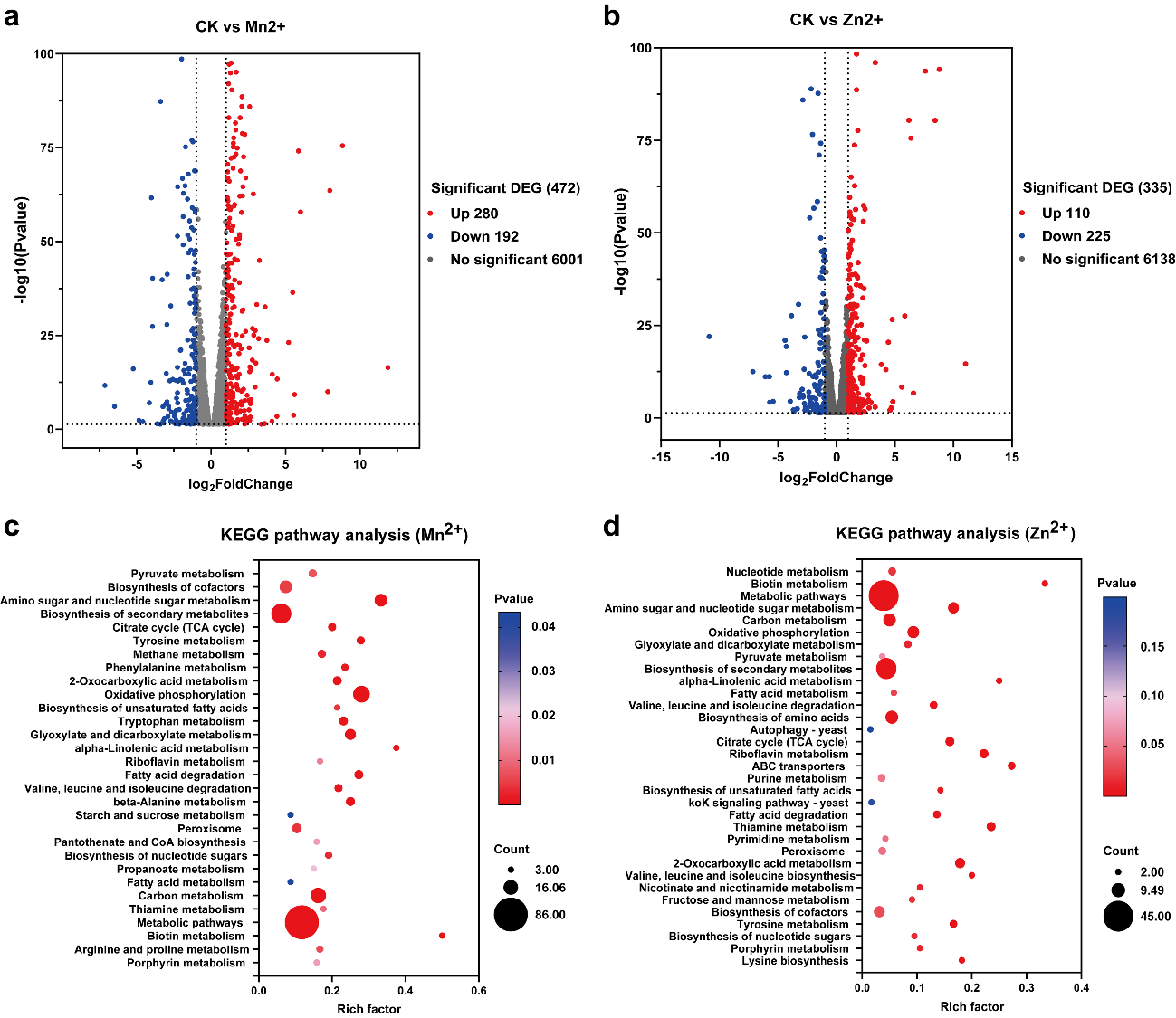


**Figure S6.** Transcriptomic analysis and annotation of DEGs between the Mn^2+^ and Zn^2+^ group. a, Volcano plot of the DEGs in Mn^2+^ group. b, Volcano plot of the DEGs in Zn^2+^ group. Each point represents a gene, the red dot indicates a significantly up-regulated genes (Log_2_FC>1), while the blue dot represents the significantly down-regulated genes (Log_2_FC<−1) in both a and b. c, KEGG enrichment analysis of DEGs in Mn^2+^ group. d, KEGG enrichment analysis of DEGs in Zn^2+^ group. The size of the dot indicates the number of differently expressed genes in the pathway and the color of the dot indicates to different Q-value ranges in c and d.





**Figure S7.** The effect of different pH values on the fed-batch fermentation of YMA-23 strain in a 5 L bioreactor. a, b, c) Growth, glucose or glycerol consumption and MA production of the YMA-23 strain under the condition of pH4, pH5 and pH6, respectively. The data were presented as mean values ± SD from three independent biological replicates (n = 3).


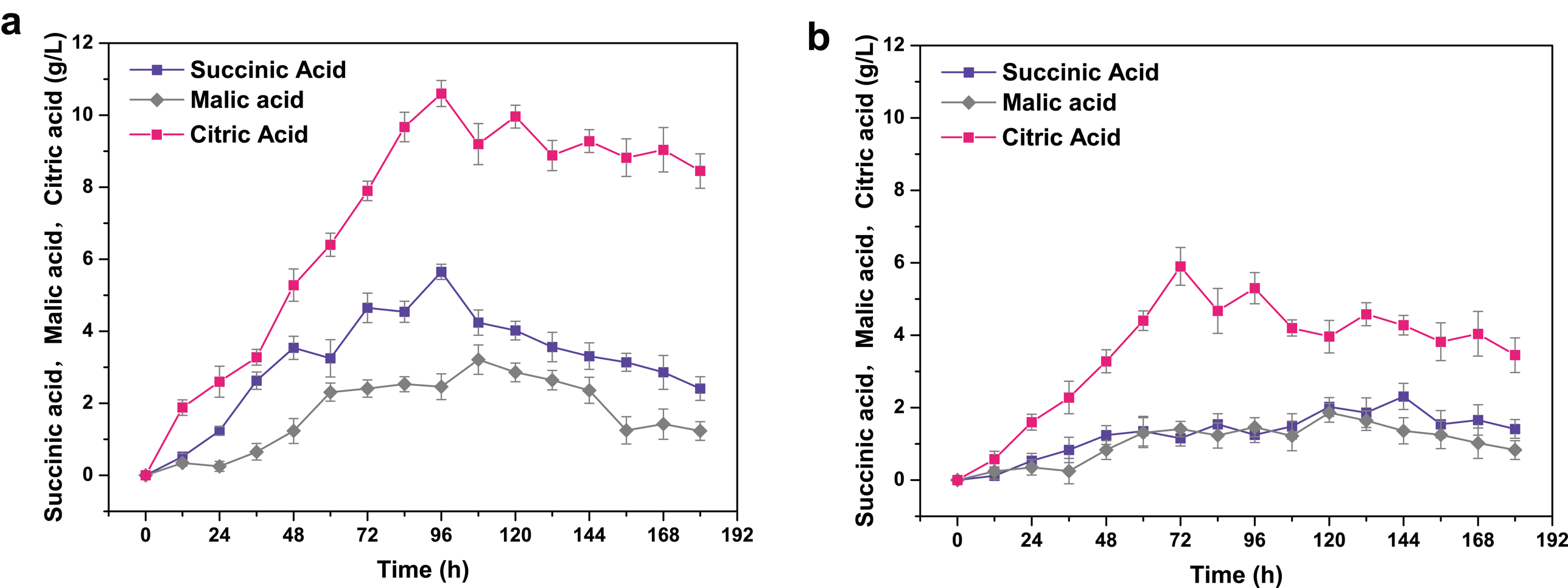


**Figure S8.** Detection and analysis of the by-products in fed-batch feeding (a) and constant-rate feeding strategies (b). The data were presented as mean values ± SD from three independent biological replicates (n = 3).

## Table S1. Strains and plasmids used in this study

| **Strains** | **Characteristics** | **Source** |
| --- | --- | --- |
| *E. coli* JM109 | Cloning host | Invitrogen |
| Po1f | *MatA, Leu2−270, Ura3−302, Xpr2−322, Axp1−2* | [1] |
| YMA-1 | *Po1f∷p1269-ScEHD3*** | This study |
| YMA-2 | *Po1f∷p1269-MDC* | This study |
| YMA-3 | *Po1f∷p1269-McrC* | This study |
| YMA-4 | *Po1f∷p1269-MDC-YneI* | This study |
| YMA-5 | *Po1f∷p1269-McrC-YneI* | This study |
| YMA-6 | *Po1f∷P_ut8_- ScEHD3**-T_cyc1_, LoxP-Leu-LoxP* | This study |
| YMA-Sc | *Po1f∷p1269-ScEHD3*** | This study |
| YMA-Yl | *Po1f∷p1269-YlEHD3*** | This study |
| YMA-Ca | *Po1f∷p1269-CaEHD3*** | This study |
| YMA-An | *Po1f∷p1269-AnEHD3*** | This study |
| YMA-Ls | *Po1f∷p1269-LsEHD3*** | This study |
| YMA-Fo | *Po1f∷p1269-FoEHD3*** | This study |
| YMA-7 | YMA-6*∷P_TEFin_-FoEHD3**-T_cyc1_, LoxP-Ura-LoxP* | This study |
| YMA-8 | YMA-7 *∆ Ku70* | This study |
| YMA-9 | YMA-8 *∆DGA1, LoxP-Ura-LoxP* | This study |
| YMA-10 | YMA-8 *∆DGA2, LoxP-Ura-LoxP* | This study |
| YMA-11 | YMA-8 *∆DGA1, P_hp4d_-POX1-T_xpr2_, LoxP-Ura-LoxP* | This study |
| YMA-12 | YMA-8 *∆DGA1, P_hp4d_-POX2-T_xpr2_, LoxP-Ura-LoxP* | This study |
| YMA-13 | YMA-8 *∆DGA1, P_hp4d_-POX3-T_xpr2_, LoxP-Ura-LoxP* | This study |
| YMA-14 | YMA-8 *∆DGA1, P_hp4d_-POX4-T_xpr2_, LoxP-Ura-LoxP* | This study |
| YMA-15 | YMA-8 *∆DGA1, P_hp4d_-POX5-T_xpr2_, LoxP-Ura-LoxP* | This study |
| YMA-16 | YMA-8 *∆DGA1, P_hp4d_-POX6-T_xpr2_, LoxP-Ura-LoxP* | This study |
| YMA-17 | YMA-8 *∆DGA1, P_hp4d_-MFE1-T_xpr2_, LoxP-Ura-LoxP* | This study |
| YMA-18 | YMA-8 *∆DGA1, P_hp4d_-POT1-T_xpr2_, LoxP-Ura-LoxP* | This study |
| YMA-19 | YMA-8 *∆DGA1, P_hp4d_-FAA1-T_xpr2_, LoxP-Ura-LoxP* | This study |
| YMA-20 | YMA-8 *ΔDGA2, P_hp4d_-FAA1-T_xpr2_,LoxP-Ura-LoxP* | This study |
| YMA-21 | YMA-9 *ΔDGA2, P_hp4d_-FAA1-T_xpr2_,LoxP-Ura-LoxP* | This study |
| YMA-22 | *YMA-21∷P_TEFin_-YlACC1-T_cyc1_* | This study |
| YMA-23 | *YMA-21∷P_TEFin_-YlACC1**-T_cyc1_* | This study |
| YMA-24 | *YMA-21∷P_TEFin_-ScACC1**-T_cyc1_* | This study |
| **Plasmids** |  |  |
| pINA1269 | expression vectors, Amp^R^ | [2] |
| pKi-1 | expression vectors, Amp^R^ | [3] |
| pKi-2 | expression vectors,Amp^R^ | [3] |
| p1269-MDC | pINA1269 carrying *MDC* gene, Amp^R^ | This study |
| p1269-McrC | pINA1269 carrying *McrC* gene, Amp^R^ | This study |
| p1269-MDC-YneI | pINA1269 carrying *MDC* and *YneI* genes, Amp^R^ | This study |
| p1269-McrC-YneI | pINA1269 carrying *McrC* and *YneI* genes, Amp^R^ | This study |
| p1269-ScEHD3*** | pINA1269 carrying *ScEHD3*** genes, Amp^R^ | This study |
| p1269-YlEHD3** | pINA1269 carrying *YlEHD3*** genes, Amp^R^ | This study |
| p1269-CaEHD3** | pINA1269 carrying *CaEHD3*** genes, Amp^R^ | This study |
| p1269-AnEHD3** | pINA1269 carrying *AnEHD3*** genes, Amp^R^ | This study |
| p1269-LsEHD3** | pINA1269 carrying *LsEHD3*** genes, Amp^R^ | This study |
| p1269-FoEHD3** | pINA1269 carrying *FoEHD3*** genes, Amp^R^ | This study |

## Table S2. The DNA sequences of the six *EHD3*** genes used in this study.

| **Genes** | **Sequence (5’- 3’)** |
| --- | --- |
| ***ScEHD3***** | ATGCTGGCCAACACCCTGGCCTGTGCTCAGCTGTCTTCTGCCTACGGCTTCGCCACCACTACCGCCACCTTCATGACCACTCAGCCTCAGCTCAACGTGACCGACGCCCCCCCCGTGCTGTTCACCGTGCAAGACACCGCCCGAGTGATCACCCTGAACCGACCCAAGAAACTGAATGCTCTGAACGCCGAGATGTCTGAGTCTATGTTCAAGACCCTGAACGAGTACGCCAAGTCTGACACCACCAACCTGGTGATCCTGAAGTCTTCTAACCGACCCCGATCTTTCTGTGCCGGCGGCGACGTGGCCACCGTGGCCATCTTCAACTTCAACAAGGAGTTCGCCAAGTCTATCAAGTTCATCACCGACTCTTACTCTCTGAACTTTCAGATCGCCACCTACCTGAAGCCCATCGTGACCTTCATGGACGGCATCACCATGGGCGGAGGCGTGGGCCTGTCTATCCACACCCCCTTCCGAATCGCCACCGAGAACACCAAGTGGGCCATGCCCGAGATGGACATCGGCTTCTTCCCCGACGTGGGCTCTACCTTCGCCCTGCCCCGAATCGTGACCCTGGCCAACTCTAACTCTCAGATGGCCCTGTACCTGTGTCTGACCGGCGAGGTGGTGACCGGCGCCGACGCCTACATGCTGGGCCTGGCCTCTCACTACGTGTCTTCTGAGAACCTGGACGCCCTGCAGAAGCGACTGGGCGAGATCTCTCCCCCCTTCAACAACGACCCTCAGTCTGCCTACTTCTTCGGCATGGTGAACGAGTCTATCGACGAGTTCGTGTCTCCCCTGCCCAAGGACTACGTGTTCAAGTACTCTAACGAGAAGCTGAACGTGATCGAGGCCTGTTTCAACCTGTCTAAGAACGGCACCATCGAGGACATCATGAACAACCTGCGACAGTACGAGGGCTCTGCCGAGGGCAAGGCCTTCGCCCAAGAGATCAAGACCAAGCTGCTGACCAAGTCTCCCTCTTCTCTGCAGATCGCCCTGCGACTGGTGCAAGAGAACTCCCGAGACCACATCGAGTCTGCCATCAAGCGAGACCTGTACACCGCCGCCAACATGTGTATGAACCAAGACTCTCTGGTGGAGTTCTCTGAGGCCACCAAGCACAAGCTGATCGACAAGCAGCGAGTGCCCTACCCCTGGACCAAGAAGGAGCAGCTGTTCGTGTCTCAGCTGACCTCTATCACCTCTCCCAAGCCCTCTCTGCCCATGTCTCTGCTGCGAAACACCTCTAACGTGACCTGGACTCAGTACCCCTACCACTCTAAGTATCAGCTGCCCACCGAGCAAGAGATCGCCGCCTACATCGAGAAGCGAACCAACGACGACACCGGCGCCAAGGTGACCGAGCGAGAGGTGCTGAACCACTTCGCCAACGTGATCCCCTCTCGACGAGGCAAGCTGGGCATTCAGTCTCTGTGTAAGATCGTGTGTGAGCGAAAGTGTGAAGAGGTGAACGACGGCCTGCGATGGAAGCACCATCACCATCACCACTAA |
| ***YlEHD3***** | ATGCTGCGAACCCTGGCCCGAACCCGAATGCCCCTGCGAGCTCAGATCAACACCCGATTCATGTCTCAGACCACCGCCGCTGCCGAGGAGTCTCCCGTGCTGTTCTCTGACAACGGCACCACCCGAACCATCACCCTGAACCGACCCAAGAAACTCAACGCCCTGGACGACCCCATGTGTCGAGCCATCTTCCCCCGACTGAAGGAGTGGCTGAAGTCTGACTCTGCCAACGTGGTGCTGTTCAAGGGCTCTGGCAATAAAGCCTTCTGTGCCGGAGGCGACGTGGCTACCCTGGCCAAGCAGAACGCCGCTGGCGCCCAAGGCATCGAGCAGTCCCGAGACTTCATCTGTACCTCTTACGCCATGGACTTCCTGCTGTCTGTGTACCCTAAGCCCGTGATCGCCCTGAACCACGGCATCACCATGGGCGGAGGCCTGGGCGTGTCTATGCACCTGCCCTTCCGAGTGGCCACCGAGACCACCCTGTCTGCCATGCCCGAGACCTCTATCGGCTTCTTCTGTGACGTGGGCGGCACCTTCTTCCTGCCCCGACTGGACGGCGAGCTGGGCACCTTCCTGGCCCTGACCTCTTCTCGACTGAAGGGCTACGAGGCCGTGGCCGCCGGCTTCGCCACCCACTACATCCCCTCTGCCCGACTCGACGACCTGGAGAAGGCCATCGCCTCTCTGGACATCCCCAAGGGCCGAGACGCCACCGAGCTGTACTCTCAGCTCAACGCTCTGCTGAACGACTACTCTGAGGTGCCCGCCAACTTCAAGTTTCAGTACGGCGGCGAGTTCCGAGCCCTGATCGACCGAACCTTCAAGTACGACGCCATCGAGGACATCGTGGCCGCCCTGAAGCAAGAGGGCGAGGCCGGCGAGAAGATCATCAAGCTGCTGGGCGAGCGATCTCCCACCTCTGTGAAGGTGACCCTGGCCGCCCTGCGACGAGGCCGAAACCAAGACATTCAGTCTGTGTTCAACGACGAGTGTAACTACGCCGAGAACTTCATGCACTCCCGAGAGTTCATCGAGGGAGTGACTGCTAAGCTGATCGACAAGCCCGCCCGAGAGCCTCAGTGGGAGCCCTCTTCTTTCGAGGGCGTGACCCCCGAGATCGTGAAGTCTTTCATTCAGAAGCGACCCGGCTCTAAGAACGACGAGATGGAGGTGGTGATCAACGACACCTACAAGCAGTACCCCTGGAACTTCGGCCTGCCCCGAGAGGTGGAGATCGAGCAGTACATCAAGGGCGAGACCCGATCTTCTGACTACAAGGCCTCTCCCGCCGAGGTGGTGAACCACTTCACCAACAAGTACTCTGGCAAGCCCGGCGTGTCTGAGAAGGTGAAAGAGGTGATGCAGCGAAAGACCAAGCCCGACCCCAACGAGCCCAAGGTGCTGGACTGGGTGAACCACCATCACCATCACCACTAA |
| ***CaEHD3***** | ATGCTGCGACTGAACAACTCTATCTCTCTGCTGAAGCAAGTGCGAAAGATCGCCACCACCTCTATCAACATGTCTTCTAAGCTGTCTACCAACCACACCTCTGGCGGCGAGGAGCCCGTGGTGCTGTCTTCTGTGAAGAACCACGCCCGACTGATCACCCTGAACCGAGTGAAGAAGCTGAACTCTCTGAACACCGAGATGATCGAGCTGATGACCCCTCCCATCCTGGAGTACGCCAAGTCTAAGGAGAACAACGTGATCATCCTGACCTCTAACTCTCCCAAGGCCCTGTGTGCCGGCGGCGACGTGGCCGAGTGTGCCGTGCAGATCCGAAAGGGCAACCCCGGCTACGGCGCCGACTTCATCGACAAGTCTTACAACCTCAACTACATCATCTCTACCCTGCCCAAGCCCTACATCTCTCTGATGGACGGCATCACCTTCGGCGGAGGCGTGGGCCTGTCTGTGCACGCCCCCTTCCGAGTGGCCACCGAGAAGACCAAGCTGGCCATGCCCGAGATGGACATCGGCTTCTTCCCCGACGTGGGCACCACCTTCTTCCTGCCCCGACTGAACGACAAGCTGGGCTACTACGTGGCCCTGACCGGCTCTGTGCTGCCTGGCCTGGACGCCTACTTCGCCGGCTTCGCCACCCACTACATTAAGTCTGAGAAGATCCCTCAGCTGATCAACCGACTGGCCGACCTGCAGCCCCCCGCCATCGAGGACGACATCACCGTGCTGTCTGGCAACAATCAGTACTTCAACCAAGTGAACGACATCCTGAACGACTTCTCTGAGAAGAAGCTGCCCGAGGACTACAAGTTCTTCCTGTCTACCGAGGACATCGCCACCATCAACAAGGCCTTCTCTCAAGACACCATCGACGACGTGCTGAAGTACCTGGAGAACGACGGCTCTCCCTTCGCCCGAAAGACCCTGGAGACCCTGCTGAAGAAGCCCAAGTCTTCTCTGGCCGTGGGCTTCGAACTGATGAATCACGGCGCCAAGAACTCTATCAAGAAGCAGTTCGAGCTGGAGATGGTGTCTGCCACCAACATCATGTCTATCCCCGCCGAGAAGAACGACTTCGCCAAGGGCGTGATCCACAAGCTGGTGGACAAGATCAAGGACCCCTTCTTCCCCAAGTGGTCTGACCCCTCTACCGTGACTCAGCAGTTCCTGTCTAACATCCTGTCTACCTCTAAGAACACCGACAAGTACCTGAAGACCCCCTTCATCAAGAAGTGGTTCGGCGTGGACTTCGAGGACTACCCCCATCAGATGGGCCTGCCCACCAACAAGCAAGTGGCCGACTACATCTCTGGCTCTGACGGCTCTAACCGAACCTACCTGCCCACCCCCGCCGAGGTGTTCAAGCACTTCAAGCAGAAGACCAACAACAAGCTGGGCGTGGACGAGAAGATCAAGCAGATCCTGGACCTGCACGGCGAGACCGCCAAGTACGACCACAAGTACGTGACCTGGAAGGAGGAGCCCACCAAGCACCATCACCATCACCACTAA |
| ***AnEHD3***** | ATGCCCCTGCGAGCCAAGCTGACCAACCCCGCCTTCGGCGCCACCGCCTCTATGTCTACCGCCCCCATCCCCAAGGAGCTGCCCGGCGACGAGCCCGACGACGTGCTGTTCCACTCTCACTACGGCGTGCGACTGATCGAGCTGAACCGACCCAAGAAGCTGAACTCTCTGAACGGCTCTATGGTGCGAAAGATCGTGCCCCGACTGAAGGAGTGGGAGAAGTCTGACCTGGCCAACGTGATCATGCTGTCTGGCGCCGGCTCTAAGGCCCTGTGTGCTGGAGGCGACGTGGCCGCTCTGGCCCTGCAAAACGAGAAGGGCCCCGAGGGACAGCAAGCCTCTTCTGACTTCATCGCCGACTCTTACCGACTGGACCACCTGATCGCCACCTATCAGAAGCCCTTCGTGTCTGTGATGGACGGCATCACCATGGGCGGAGGCGTGGGCCTGTCTGTGCACGCCCCCTTCCGAATCGCCACCGAGCGAACCGTGTTCGCCATGCCCGAGACCACCATCGGCTTCTTCCCCGACGTGGGCGGCTCTTTCTTCCTGTCTCGACTGGACGGCGAGCTGGGCACCTACCTGGCCCTGACCTCTGAGCGACTGCACGGCGTGCAAGCCCTGTACGCCGGCGTGGCCACCCACTACCTGCACTCTTCTGCCCTGGCCAACCTGACCGCCCGACTGTCTGAGCTGGTGTTCCGAGACTACTCTACCTTCCAAGACCGACTGGCCCTGGTGAACAAGACCATGGCCGAGTTCTCTACCGGCGTGCCCTCTGTGCGAGAGGAGCCCATTCAGCTGGCCGGCAAGCTGCGATCTGCCATCGACCGATGTTTTCAGTACAACACCGTGGAGGAGATCATCCAAGCCCTGCAGAAGGAGACCGAGATGAAGTCTTGGGCCGAGAAGACCCTGGAGACCCTGTCTGCCCGATCTCCCACCTCTCTGAAGGTGGCCCTGCGACAGCTGCGAGTGGGCCGACAGTGGACCATCTCTGAGACCTTTCAGCGAGAGCACGCCATCGCCTCTAAGTTCATGCGACACCCCGACTTCGTGGAAGGCGTGAAGGCCCGACTGATGTCTAAGCCCCCCCGACAAGCCACCTGGCAGCCCGCCACCCTGGAAGAGGTGTCTACCGAGGCCATCGATCAGTTCTTCGAGATCCCCGAGTCTGCCTCTGGCCCCGAGTCTCGACTGTCTCTGTACCACTACAAGTCTCCCTACACTCAGTACCCCTACAAGTTCGGCCTGCCCTCTGAGTCTCGAATCGAGGCCTTCGTGCGACACCGAGGCCGAAAGGGCGACCTGACCCTGAAGGAGATCGTGTCTAACTTCGACTCTAAGGAGGGCGTGAAGGAGAAGGTGGCCGAGGTGCTGGCCCGACGAACCGTGCGAGACGAGGCCGGCCTGCACTGGGTGAACCACCATCACCATCACCACTAA |
| ***LsEHD3***** | ATGCCCCTGCGAGCCAAGATCACCAACCCCGCCTTCGCCGGCTCTAAGGCCCACATGTCTACCTCTGAGGGCCCCGAAGTCATCCGAGAGCTGCCCGGCGACGAGAAGGAGGACGTGCTGTTCGACACCCTGTTCGGCCTGCGAACCATCGAGCTGAACCGACCCGCCAAGCTGAACTCTCTGAACGGCTCTATGATCCGAAAGATCCTGCCCCGACTGCAAGAGTGGGCCAAGTCTGACATGGCCAACGTGGTCGTGATCAAGGGCCAAGGCCCCAAGGCCTTCTGTGCCGGCGGCGACGTGTCTTCTCTGGCCATCGACAACACCAAGGGCGAGGAGGGACAGAAGCGATCTTCTGACTACATCGCCCTGTCTTACAAGCTGGACCACCTGATCGCCACCTACACCAAGCCCTACGTGGCCTTCATGGACGGCATCACCATGGGCGGAGGCGTGGGCCTGTCTGTGCACGCCCCCTTCCGAATCGCCACCGAGAAGACCGTGTTCGCCATGCCCGAGACCACCATCGGCTTCTTCCCCGACGTGGGCGCCTCTTTCTTCCTGCCCCGAATGGCCGGCGCCACCGGCACCTACCTGGCCCTGACCTCTGAGAAGCTGAAGGGCGTGGACGCCTACTACGCCGGCATCGCCACCCACTACGTGCACTCTACCTCTCTGGCCGCCCTGGAGCGACGACTGGCCGAGCTGCGATTCAAGGACTACGACTCTCTGGACGTGCGACTGAACCTGATCGACTCTACCATCGAGGAGTTCGCCACCGGCCTCCCCCACGATCAGCCCATGATGCTGTCTGGCAAGATCCGAGAGGCCATCGACTACTGTTTCGGCGCCGACTCTGTGAAGCAGCTGTACGGCCGACTGAAGGACGACAAGTTCCGAGGCGCCGAGCCCAAGCCCGAGGTGATTGGCACCAACGAGGAGACCGGCGAGGAGATCTGGTCTAAGAAGGGCCCCGTGACCACCATGCTGGACAACACCCTGGAGGCCCTGGAGAAGCGATCTCCCACCTCTCTGTACGTGGCTTTCCGACAGATGAAGCTGGGACAGAACTGGTCTATCGCCGAGACCTTTCAGCGAGAGCATCAGATGGCCTCTAAGTTCATGCGACACAACGACTTCACCGAGGGCGTGCACGCCCTGCTGATCCGAAAGGACGGCAAGCCCAAGTGGGACCCCCTGCCCGACGAGCCCGAGGAGCGAGAGCGAGTGGCCACCTCTTTCTTCGAGGTGGAGGGCGAGCAGCGACTGAAGCTGCTGACCGGCACCGACTACAAGGAGTACCCCCACTGGCGATTCGGCCTGCCCCAAGAGAAGCACATCGAGGCCGTGGTGAACGAGGGCGACAAGACCGTGGCCGAGGTGGTGGAGTACTTCGTGAAGGCCAAGCAAGGCAAGCAAGGCGTGAAAGAGGTGGTGTCTGAGGTGCTGGCCCGAAGAGCCCACTCCGGCGACAACGGCAAGGCCTCTTGGCGAAAGTCTAAGAAGGCCGCCCACCATCACCATCACCACTAA |
| ***FoEHD3***** | ATGGTGCAGCAGTCTAAGATCCTGTCTTCCTCTCTGGGCGCCCGACAGCTGTCTTCTGAGGCCACCAACATCCGAGAGAAGCGAGGCGACGACCCCAACGACGTGGTGTTCGAGTCTAAGTACGGCCTGCGAACCGTGATGCTGAACCGACCTCAGAAGCTGAACTCTCTGAATGCCTCTATGATCCGAAAGATCGTGCCCCGACTGATCGAGTGGGAGAAGTCTGACCTGGCCAACGTGGTCGTGATGAAGGGCGCTGGCGAGAAGGCTCTGTGTGCCGGCGGCGACGTGGCCGCCCTGGCCAAGCTGAATTCTCGATCTGAGGACGGCTGGCAGAAGTCTGCTCAGTACATCGCCCTGTCTTACAAGCTGGACCACTACATCGCCACCTACAAGAAGCCCTACATCGCCTTCATGGACGGCATCACCATGGGCGGAGGCGTGGGCCTGTCTGCCCACGCCCCCTTCCGAATCGCCACCGAGAAGACCGTGTTCGCCATGCCCGAGACCACCATCGGCTTCTTCCCCGACGTGGGCGCCTCTTTCTTCCTGCCCCGAATGAACGGCTCTGTGGGCACCTACCTGGCCCTGACCTCTGAGCGACTGACCGGCCCCAACGTGTTCTACTCTGGCATCGCCACCCACTACCTGCACTCTACCTCTCTGCCCGACCTGGAGGCCCGACTGGCCGAGCTGCGATTCCGAGACTCTGACGGCCTGCCCGAGCGACTGGCCCTGATCAATCAGACCCTGGAGGAGTTCTGTACCGGCCTGCCCTACGATCAGCCCATCACCCTGTCTGGCGAGATCCGACAAGCCATCGACCGATGTTTCAACAAGCACACCATCTCTGAGATCATTGCCGCTCTGCAAGCCGAGCGAGGCCCCACCGAGGAGTGGGCTCAGAAGCAGCTGAAGACCCTGCACAAGCGATCTCCCACCGCCGTGCACGTGGCCCTGCGACAGATGCGAGTGGGCGGCGAGTGGGACATCGCCGAGACCTTCAAGCGAGAGCATCAGATCGCCACCAAGTTCATGCAGCACCCCGACTTCACCGAGGGCGTGTCTGCCCTGCTGATCCGAAAGGAGGCCCCCAAGTGGCAGCCCGAGTCTCTGGAGGCCATCGGCGGCACCAACGTGGCCAAGCCCTTCTTCGAGTACGACTCTAACAACGAGCTGGCCCTGTTCACCGACCGAACCTTCAAGGAGTACCCCCACCGAGAGCTGGGCGTGCCCTCTGAGAAGGAGATCGAGCAAGTGCTGTCTTCTGGCACCTACACCCAAGAGCAGCTGGCCAACAAGATCGTGTCTTCTCGAAACGGCCGACAAGGCATCGCCGAGGTGGCCCGAGAAATCATCGCCCGAAAAACCGCCGTGGACGACCAAGGCAAGGCCGTGTGGATGGCCGACGAGTCTCTGCCCGGCTCTCGACTGCACCATCACCATCACCACTAA |

## Table S3. Primers used in this study

| **Primers** | **Sequence (5’- 3’)** |
| --- | --- |
| P1269-F | ggatccTTAGTTTCGGGTTCCcacg |
| P1269-R | ggatccAACTACGGAACTTGTGTTG |
| ScEHD3-F | cgtgGGAACCCGAAACTAAggatccATGCTGGCCAACACCCTGGCCT |
| YlEHD3-F | acgtgGGAACCCGAAACTAAggatccATGCTGCGAACCCTGGCCCGA |
| CaEHD3-F | cgtgGGAACCCGAAACTAAggatccATGCTGCGACTGAACAACTCTATC |
| AnEHD3-F | cgtgGGAACCCGAAACTAAggatccATGCCCCTGCGAGCCAAGCTGAC |
| LsEHD3-F | cgtgGGAACCCGAAACTAAggatccATGCCCCTGCGAGCCAAGATCAC |
| FoEHD3-F | cgtgGGAACCCGAAACTAAggatccATGGTGCAGCAGTCTAAGATCCTG |
| EHD3-R | CAACACAAGTTCCGTAGTTggatccTTAGTGGTGATGGTGATGGTG |
| PKI1-F | TAATCATGTAATTAGTTATGTCAC |
| PKI1-R | GAGCATCTTTGAATGATTCTTATACTCA |
| PKI2-F | CGTGGATGTGTGTGGTTGTAT |
| PKI2-R | GTACCTCCATGGCCTGTCCCCA |
| McrC-F | AACTAATTACATGATTAAACAGTAATAGCTCTACCTCTA |
| McrC-R | agtactaaccgcagATGTCTGCTACTACTGGTGCTAG |
| MDC-F | AACTAATTACATGATTAACCACACATAAAATAAAATCTC |
| MDC-R | agtactaaccgcagATGGGTCCATTTCCATTGTCTTCAG |
| YneI-F | ACACACATCCACGATGACTATAACCCCGGCAACCCAC |
| YneI-R | ACAGGCCATGGAGGTACTCAGATACGGTCCTTCCATAC |
| DGA1-UHA-F | ATGCTGCGGGCGGATCCTGGT |
| DGA1-UHA-R | GTTATCCGAAGCGATAGCTTTTGTTTTGTGTGACTTGTC |
| DGA1-DHA-F | TGCTATACGAAGTTATGGAAAACTGCCTGGGTTAGGCA |
| DGA1-DHA-R | TCTGATGGCCTGGAGCGAGTTTC |
| DGA2-UHA-F | TGGGAGTGTATTTGGAAAATGACT |
| DGA2-UHA-R | GTTATCCGAAGCGATTTTGCGGGCGGTACGGGTACAGC |
| DGA2-DHA-F | CTATACGAAGTTATCATAACACTCATCAGTAGCCTTTAC |
| DGA2-DHA-R | CGAAACGCATTAGAAGTAATTAGC |
| FAA1-F | ACTAATTACATGACTAAGACTGCTCGTAGCACTCATC |
| FAA1-R | CCACACACATCCACGATGGTCGGATACACAATTTCCTCA |
| POX1-F | CATAACTAATTACATGATCACTCATCGAGATCGCAAATttc |
| POX1-R | ACACACATCCACGatggccaaggagcGAGGTAAGACT |
| POX2-F | ACACACATCCACGATGAACCCCAACAACACTGGCAC |
| POX2-R | ACTAATTACATGACTATTCCTCATCAAGCTCGCAAATG |
| POX3-F | CACACACATCCACGATGATCTCCCCCAACCTCACAGCT |
| POX3-R | ACTAATTACATGACTATTCCTCGTCCAGCTCGCAAATG |
| POX4-F | ACACACATCCACGATGAACAACAACCCCACCAACGT |
| POX4-R | ACTAATTACATGACTACTCGTCCAGGTCGCAAATCTC |
| POX5-F | CACACACATCCACGATGCTCTCTCAACAGTCCCTCA |
| POX5-R | ACTAATTACATGACTACTCATCCTCAAGAGAGCA |
| POX6-F | CACACACATCCACGATGATCACCCCAAACCCCGCTAAC |
| POX6-R | AACTAATTACATGATTACTGAATATCCTCGGGCTCCAT |
| MFE1-F | ACACACATCCACGAAGTGCTAAAAAATCACGTCCAACA |
| MFE1-R | ACTAATTACATGATCACTTGACCTGAATACCACACA |
| POT1-F | CACACACATCCACGATGAACCACTACTCACACAACCCT |
| POT1-R | ACTAATTACATGATTAGTGCTTGAGAGTCTCGTTCTTG |
| ScACC1-F | ctttcttcgctcatctgcggttagtactgcaaaaagtgc |
| ScACC1-R | aaTCCAAGCTGtaaTCATGTAATTAGTTATGTCACGCTT |
| YlACC1-F | cagtactaaccgcagATGGCCACCCCCGAAGATCTCGCT |
| YlACC1-R | gctgagctgctcaagggGTTGTGATCATGTAATTAGT |

**Supplementary References**

[1] C. Madzak, C. Gaillardin, J. M. Beckerich, *J. Biotechnol.* **2004**, *109* (1-2), 63.

[2] J. M. Nicaud, C. Madzak, P. van den Broek, C. Gysler, P. Duboc, P. Niederberger, C. Gaillardin, *FEMS Yeast Res.* **2002**, *2* (3), 371.

[3] Z. Cui, Y. Zhong, Z. Sun, Z. Jiang, J. Deng, Q. Wang, J. Nielsen, J. Hou, Q. Qi, *Nat Commun* **2023**, *14* (1), 8480.
